# Phase transition and switchable dielectric behaviours in an organic–inorganic hybrid compound: (3-nitroanilinium)_2_(18-crown-6)_2_(H_2_PO_4_)_2_(H_3_PO_4_)_3_(H_2_O)

**DOI:** 10.1098/rsos.180738

**Published:** 2018-10-03

**Authors:** Yang Liu, Chun-li Zhu, Xiao-yuan Zheng, Liu-lei Qin, Shuang-xi Yang, Zun-qi Liu

**Affiliations:** Chemical Engineering College, Xinjiang Agricultural University, Urumqi 830052, People's Republic of China

**Keywords:** crystal structure, structure phase transition, dielectric anisotropy, pendulum-like motion, three-dimensional layer

## Abstract

An organic–inorganic hybrid compound with an extensive three-dimensional (3D) crystal structure, (3-nitroanilinium)_2_(18-crown-6)_2_(H_2_PO_4_)_2_(H_3_PO_4_)_3_(H_2_O) (**1**), was synthesized under slow evaporation conditions. Differential scanning calorimetry measurements indicated that **1** underwent a reversible phase transition at *ca* 231 K with a hysteresis width of 10 K. Variable-temperature X-ray single-crystal diffraction revealed that the phase transition of **1** can be ascribed to coupling of pendulum-like motions of its nitro group with proton transfer in O–H···O hydrogen bonds of the 3D framework. The temperature dependence of its dielectric permittivity demonstrated a step-like change in the range of 150–280 K with remarkable dielectric anisotropy, making **1** a promising switchable dielectric material. Potential energy calculations further supported the possibility of dynamic motion of the cationic nitro group. Overall, our findings may inspire the development of other switchable dielectric phase transition materials by introducing inorganic anions into organic–inorganic hybrid systems.

## 1. Introduction

Phase transition compounds with electric, magnetic, and thermally switchable ON/OFF states usually provide an efficient strategy for the design of multifunctional switching materials such as ferroelectrics [1–9]. Generally, such physical responses always exhibit abrupt changes in the vicinity of a phase transition, giving rise to phase transition materials with potential applications in energy storage, temperature controlling, data communications and phase shifters [10–19]. Molecular switchable dielectrics typically have dielectric constants that vary between high and low states upon temperature change. For this reason, they are one of the most attractive and promising phase transition materials for application in modern multifunctional materials [20–26]. In this regard, organic–inorganic hybrid compounds that combine desirable characteristics from both types of constituents, especially those that can be prepared into large-size crystals, may be an effective strategy for obtaining ideal phase transition materials [27–33].

Several novel organic–inorganic hybrid phase transition materials with electrical switches have been synthesized [34–38]. Previous studies have also demonstrated that freezing and reorientation of the organic cations and deformation of the anionic framework can easily induce phase transitions. To obtain molecular crystals that exhibit such phase transitions, complexes based on crown ether and its derivatives have recently been investigated [39–46]. These materials are crucial not only because they comprise diverse molecule motions, thereby providing favourable conditions for reversible phase transitions; but also for their extensive utilization in supramolecular chemistry and crystal engineering for assembling multiple ionic compounds to produce an assortment of host–guest molecules [47–49]. Tang *et al*. successfully demonstrated a class of perovskite structure clathrates, [Hcpa-(18-crown-6)]^+^[ClO_4_]^−^, where Hcpa represents the protonated cyclopentylamine cation, that display unusual multisequential reversible phase transitions accompanied by switchable dielectric behaviour. Sequential reversible phase transitions and symmetry breaking occurs in response to the stepwise synergistic disordering of the Hcpa cations and ClO_4_^−^ anions. Shi and co-workers reported the supramolecular phase change material (PCM) potassium hydrogen bis(dichloroacetate)-18-crown-6, which undergoes a reversible second-order phase transition at 181.8 K (*T*c). The phase transition was ascribed to order–disorder transformations of pendulum-like motions of the CHCl_2_COO/CHCl_2_COOH units between the low- and room-temperature phases [50–52].

We have previously reported molecular rotators based on supramolecules formed by crown ether and anilinium derivatives that showed reversible phase transitions via hydrogen bonding. For example, a layered crystal of (2-nitroanilinium)(18-crown-6)(IO_4_) showed a pair of reversible peaks at 260.5 K (cooling) and 264.5 K (heating) with heat hysteresis of approximately 4 K. Structural analysis and potential energy calculations indicated that the dielectric anomaly and phase transition occur because of intra- and intermolecular proton transfer of −NH_3_^+^ in 2-nitroanilinium as a consequence of –NO_2_ motion in the cation [53–55]. It was suggested that hydrogen-bonding interactions and/or molecular motion give rise to symmetry breaking, thus leading to the dielectric anomaly behaviour as well as the phase transition. With a view to expand this research, a protonated 3-nitroanilinium cation that can form supramolecular rotator structures with 18-crown-6 through hydrogen-bonding interactions was adopted as a new type of molecular phase transition material. Compared with 2-nitroanilinium, 3-nitroanilinium provides a larger volume for molecular motion within the crystal, thereby reducing the potential energy required for molecular motion. Meanwhile, H_2_PO_4_^−^ is used as the counter-ion in the supramolecular cation assembly. By providing active H atoms, H_2_PO_4_^−^ easily forms hydrogen-bonding interactions that greatly contribute to the phase transition behaviour and dielectric and ferroelectric properties of the material. In this study, we successfully synthesized a new organic–inorganic hybrid compound, (3-nitroanilinium)_2_(18-crown-6)_2_(H_2_PO_4_)_2_(H_3_PO_4_)_3_(H_2_O) (**1**), which not only shows sequential reversible structure phase transition at *ca* 231 K, but also exhibits switchable dielectric anisotropy behaviour along the *a*-, *b*- and *c*-axes. The molecular motions are discussed in terms of X-ray crystallographic analyses and potential energy calculations.

## 2. Experimental

### 2.1. Material and methods

All reagent-grade chemicals and solvents were of analytical grade and used without any further purification. The IR spectra of compound **1** were recorded on an Affinity-1 spectrophotometer (400–4000 cm^−1^) at room temperature, with all samples prepared as diluted KBr pellets (electronic supplementary material, figure S1). Elemental analysis was carried out on a Vario EL Elementar Analysen systeme GmbH at the Collaboration Center of TRW Research, Shandong. Thermogravimetric analysis (TG) and differential thermal analysis (DTA) measurements were performed on a TA Q50 instrument under flowing nitrogen at a heating rate of 10 K min^−1^. Differential scanning calorimetry (DSC) measurements were performed on a TA Q2000 DSC instrument by heating and cooling the crystal samples (12.3 mg) at a rate of 10 K min^−1^ within the temperature range of 210–260 K under nitrogen at atmospheric pressure. Single crystals of **1** deposited with a silver conducting glue were used for the dielectric measurements.

### 2.2. Preparation of **1**

**Scheme 1.**
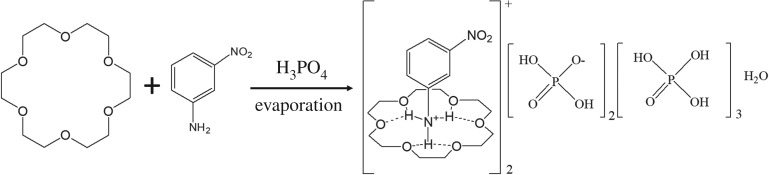
Synthesis of compound **1**.

The title compound **1** was prepared by slow evaporation of a mixture solution at room temperature, as shown in scheme 1. An acetonitrile solution (20 ml) of 3-nitroaniline (1.4 mol, 0.208 g) was added slowly to an acetonitrile solution (20 ml) of 18-crown-6 (1.4 mol, 0.400 g), followed by the dropwise addition of 0.174 g phosphoric acid (85%). The solvent was evaporated for five days at room temperature to give a block crystal of 1 in 65.5% yield. Elemental analysis calcd (%) for compound **1** (C_36_H_77_N_4_O_37_P_5_, formula weight: 1312.87): C 32.93; H 5.91; N 4.27; Found: C 33.02; H 5.83; N 4.19.

### 2.3. Single-crystal structure determination

Crystallographic data of single crystals of **1** were obtained using a Bruker AXS CCD area-detector diffractometer with Mo-Kα radiation (*λ* = 0.71073 Å) at 100 and 296 K. The data collection and cell refinements were obtained using the CrystalClear software package (Rigaku). The structures were solved by direct methods and successive Fourier synthesis and refined on *F*_2_ by the full-matrix least-squares method (SHELXTL-97). All non-hydrogen atoms were refined anisotropically using reflections with *I* > 2*δ* (*I*). Hydrogen atoms were added geometrically and refined using the riding model with Uiso(H) = 1.2Ueq. The packing views were drawn with Crystal Make, and selected bond distances and angles were calculated using SHELXTL. Table 1 provides a summary of the crystallographic data and details of the data collection and refinement. CCDC: 1569746 (100 K) and CCDC: 1569747 (296 K) for **1** contain the supplementary crystallographic data from this study. These data can be obtained free of charge from The Cambridge Crystallographic Data Centre via www.ccdc.cam.ac.uk/data_request/cif.

**Table 1.** Crystal data and structural refinements for compound **1** at 100 and 296 K.

temperature	100 K	296 K
chemical formula	C_36_H_77_N_4_O_37_P_5_	C_36_H_77_N_4_O_37_P_5_
formula weight	1312.87	1312.87
crystal size (mm^3^)	0.21 × 0.20 × 0.19	0.21 × 0.20 × 0.19
crystal system	monoclinic	monoclinic
space group	*P*2_1_/c	*P*2_1_/n
*a* (Å)	13.8655(17)	13.949(2)
*b* (Å)	25.404(3)	25.907(5)
*c* (Å)	21.834(3)	16.829(3)
*α* (°)	90.00	90.00
*β* (°)	129.423(9)	90.085(2)
*γ* (°)	90.00	90.00
*V* (Å^3^)	5941.0(13)	6081.7(18)
*Z*	4	4
*D*_calc_ (g cm^−1^)	1.468	1.433
*F*(000)	2768	2764
*m* (mm^−1^)	0.255	0.249
measured 2*q* range (°)	0.998–25.01	0.992–25.01
*R*_int_	0.0344	0.0571
*R* (*I* > 2*s*(*I*))^a^	0.0401	0.1168
_W_*R* (all data)^b^	0.1029	0.1350
GOF	1.047	1.033

^a^$R=\;\sum (\left|F_{o}|-|F_{c}\right|)/\sum |F_{a}|$.

^b^$R_{w}^{2}=\sum_{w}{\left(F_{o}^{2}-F_{C}^{2}\right)}^{2}/\sum_{w}{\left(F_{o}^{2}\right)}^{2}$.

### 2.4. Calculations

To characterize the molecular motions of the 3-nitroanilinium cation, potential energy calculations were performed at the RHF/6-31G(d) level of theory. The structural units of compound **1** used in the calculations were (3-nitroanilinium)_3_(18-crown-6)_3_(H_2_PO_4_)_2_ and (3-nitroanilinium)_3_(18-crown-6)_3_, which differ from the actual stoichiometry of the compound to make the calculations computationally tractable. These computations were based on the fixed atomic coordinates obtained from the crystal structure at 296 K. The relative energy was calculated by evaluating the rigid rotation of the C_(25)_–N_(1)_ bond axes in 30° increments (electronic supplementary material, figure S9a). The nearest-neighbour molecules around the nitro-group (–NO_2_) of the 3-nitroanilinium cation were included in the calculation of the potential energy curves (electronic supplementary material, figure S9b and S9c). The relative energy of the structure was obtained by evaluating the rigid rotation of the nitro group along the C_(27)_–N_(2)_ and C_(33)_–N_(4)_ bonds, with the rotation performed every 5° in the range of −35° to 35°.

## 3. Results and discussion

### 3.1. Spectral properties

The structure of compound **1** was characterized by IR spectroscopy (electronic supplementary material, figure S1). The IR spectrum of compound **1** shows a series of characteristic peaks at 1543, 1458 and 1355 cm^−1^, which are assigned to the skeletal vibrations of the benzene rings. The strong broad peak from 2715 to 3246 cm^−1^ indicates that the –NH_2_ group is protonated by means of intermolecular N–H···O hydrogen bonds. Characteristic peaks of the 18-crown-6 molecules are observed at 1099, 993 and 835 cm^−1^, which we attribute to their –O-C-C- structural units. The bands within the 1051–848 cm^−1^ region are characteristic of the P-O stretching vibrations in H_3_PO_4_ or H_2_PO_4_^−^. Overall, these results establish the existence of three constituents in compound **1** as 3-nitroanilinium, 18-crown-6, and H_3_PO_4_/H_2_PO_4_^−^.

### 3.2. Differential scanning calorimetry and thermogravimetric analysis

**Figure 1.**
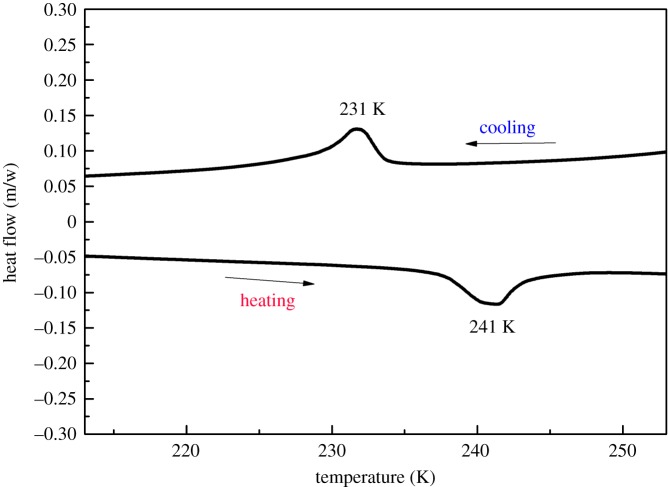
DSC curves of **1** obtained for heating–cooling experiments at a heating rate of 10 K min^−1^.

DSC is a thermodynamic method that detects the temperature dependence of reversible phase transitions. When a compound is subjected to a disorder–order transition of molecular or proton motion, reversible heat anomalies upon heating and cooling can be detected in DSC measurements. In such measurements of **1**, a pair of reversible heat anomalies at 231/241 K (cooling/heating) was clearly observed, indicating that **1** exhibits a phase transition at *T*c = 241 K (figure 1). The relatively large heat hysteresis of 10 K and the peak shapes indicate the occurrence of a reversible phase transition with a probable first-order feature. Entropy changes Δ*S* in the cooling and heating processes were estimated to be 6.32 and 8.19 J (mol K)^−1^, respectively. According to Boltzmann's equation, Δ*S* = *R*ln(*N*), where *R* is the universal gas constant and *N* is the ratio of the number of geometrically distinguishable orientations, the values of *N*_1_ and *N*_2_ are calculated as 2.14 and 2.68, respectively. These values suggest that the number of independent orientations of disordered moieties in the crystal structure of **1** changed during the phase transition, which will be confirmed by structural analysis (vide infra).

The thermal behaviour of **1** was investigated through TG and DTA measurements from 300 to 850 K (electronic supplementary material, figure S2). The DTA curve shows a relatively weak endothermic peak at 421.5 K, which corresponds to the melting point of **1**. The TG curve reveals two main weight loss regions. The structure of compound **1** remains ordered up to 421.5 K. The first weight loss comprises approximately 61.98% of the total weight, and appears to involve the loss of (3-nitroanilinium)_2_(18-crown-6)_2_(H_2_O) (calcd. at 63.54%). The second weight loss step of 38.02% (calcd. at 37.84%) involves three H_3_PO_4_ molecules and two H_2_PO_4_^−^ anions.

### 3.3. Crystal structure of **1**

**Figure 2.**
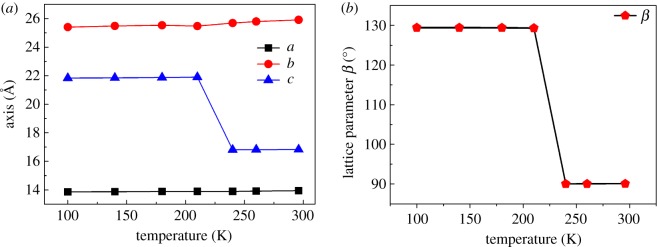
Temperature dependence of (*a*) cell parameters for three axis lengths and (*b*) *β* in the range from 100 to 296 K in **1**.

The phase transition of **1** was further confirmed at 100 K (low-temperature phase, LTP) and 296 K (room-temperature phase, RTP) by variable-temperature single-crystal X-ray structure determination. The RTP structure was solved in the monoclinic *P*2_1_/*n* space group with cell parameters of *a* = 13.949(2) Å, *b* = 25.907(5) Å, *c* = 16.829(3) Å, *β* = 90.085(2)°, *V* = 6081.7(18) Å^3^, and *Z* = 4. The LTP structure was crystallized in the same monoclinic space group *P*2_1_/*c* with cell parameters of *a* = 13.8655(17) Å, *b* = 25.404(3) Å, *c* = 21.834(3) Å, *β* = 129.423(9)°, *V* = 5941.0(13) Å^3^, and *Z* = 4. By comparison of their cell parameters and refinements data, obvious changes in the *c*-axis and *β* angle in the RTP and LTP structures are evident. First, the unit cell length *c* extends from 16.829(3) Å to 21.834(3) Å for **1**, an increase of *ca* 23%. Second, a significant change in the *β* angle from 90° to 129.423° is observed. The structural anomalies corresponding to the phase transition can be ascertained by measuring the unit cell parameters of **1** as a function of temperature from 100 to 296 K (figure 2). The lattice constants along the *a* and *b* axes show small differences between the LTP and RTP as a function of temperature. The other cell parameters, *c* and *β*, exhibit an abrupt increase near 235 K, suggesting that this phase transition is of first-order nature. Notably, this result is consistent with the results of the DSC measurements.

**Figure 3.**
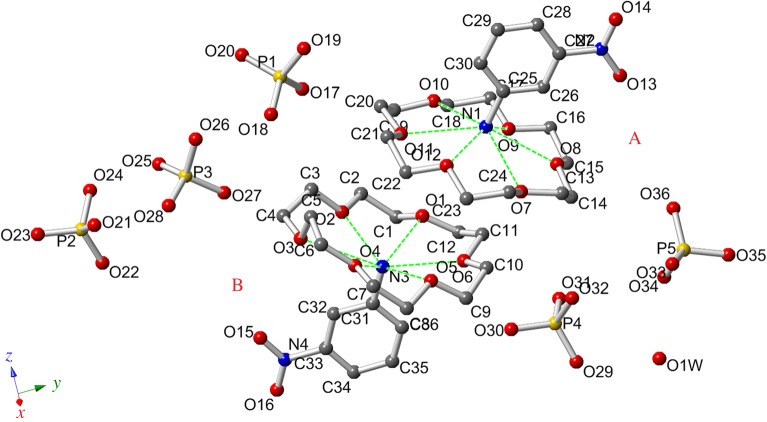
View of the asymmetric unit of compound **1** at 100 K with an atomic numbering scheme.

The asymmetric unit of **1** contains two 18-crown-6 molecules, two 3-nitroanilinium cations, two H_2_PO_4_^−^ anions, three H_3_PO_4_ molecules, and a H_2_O molecule in the LTP and RTP structures (figure 3). Although the anisotropic thermal factors of all atoms in the RTP of **1** were larger than those in the LTP, no significant changes in the crystal structure are evident (electronic supplementary material, figure S3 and table S4). The 3-nitroanilinium cation interacts with the 18-crown-6 moiety by way of six N–H···O hydrogen bonds, thereby forming two similar supramolecular cations: (3-nitroanilinium)(18-crown-6)(A) (containing atoms N_1_ and N_2_) and (3-nitroanilinium)(18-crown-6)(B) (containing atoms N_3_ and N_4_). For convenience, we have labelled the supramolecular cation A as LTP-A and RTP-A and the supramolecular cation B as LTP-B and RTP-B in the LTP and RTP structures. In LTP-A and RTP-A, a 1 : 1 [(3-nitroanilinium)(18-crown-6)]^+^ complex was formed through intermolecular −NH_3_^+^ ···O hydrogen-bonding interactions between the ammonium moiety of the 3-nitroanilinium cation and six oxygen atoms of the 18-crown-6 moiety. The average hydrogen-bonding distances involving N_1_–O were 2.900 Å (RTP) and 2.916 Å (LTP), which are almost identical to the standard NH_3_^+^ ··· ;O distance. This suggests that there was no distinct proton transfer in the N–H···O hydrogen bonds of the supramolecular cation A (electronic supplementary material, table S1). Further, the six oxygen atoms of the 18-crown-6 molecule are nearly coplanar. Indeed, the dihedral angles between the upper three (O_8_, O_10_, O_12_) and lower three (O_7_, O_9_, O_11_) oxygen planes are 1.85° at 100 K and 4.06° at 296 K, respectively. The 18-crown-6 molecules display slight distortion, with average O–C–C–O torsion angles varying from 60.38° to 68.38° in the LTP structure and from 45.15° to 68.12° in the RTP structure (electronic supplementary material, table S3).

**Figure 4.**
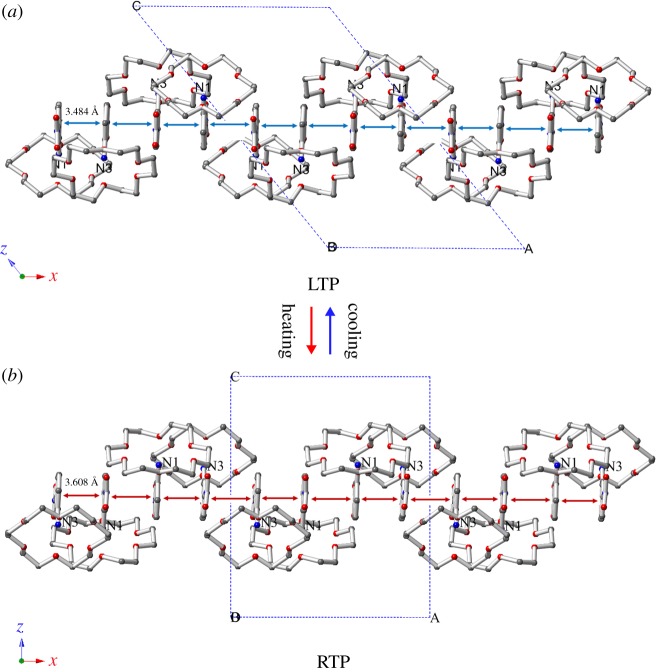
Crystal structure packing diagrams of supramolecular cations at (*a*) 100 K (LTP) and (*b*) 296 K (RTP) of **1** viewed along the *b*-axis. The dashed lines indicate *π*–*π* interactions.

The most notable differences in the structures of LTP-B and RTP-B occur for the dihedral angles between the nitro group and the aromatic ring of the 3-nitroanilinium cation (0.81° and 4.29°, respectively), which are similar to the analogous values of 1.93° and 4.89° in the LTP-A and RTP-A structures. This may be caused by intermolecular repulsion between the adjacent hydrogen atoms of the 3-nitroanilinium cations and H_2_PO_4_^−^ anions. It is clearly shown that pendulum-like motions of the nitro group can occur easily, and such motions may be the driving force for the reversible phase transition of **1** (electronic supplementary material, figure S5). The hydrogen bond interactions among the nitrogen and oxygen atoms include N_3_···O bond lengths of 2.916 Å in the RTP structure and 2.903 Å in the LTP structure. The values of the N–H···O hydrogen bond distances are similar in the RTP and LTP structures, and they are consistent with that of the supramolecular cation A. At the same time, the benzene ring Cg′ (O_25_, O_26_, O_27_, O_28_, O_29_, O_30_, N_1_, N_2_) of the supramolecular cation A is connected with the neighbouring benzene ring Cg″ (O_31_, O_32_, O_33_, O_34_, O_35_, O_36_, N_3_, N_4_) of the supramolecular cation B through *π*–*π* intermolecular interactions. The result of these interactions is formation of an unusually compact one-dimensional chain structure for LTP and RTP (figure 4 and electronic supplementary material, figure S6). By comparing the distances of the *π*–*π* interactions in the two LTP and RTP structures, an average Cg′–Cg″ distance of 3.608 Å in the RTP structure is determined, which is slightly longer than that in the LTP structure (3.484 Å, table 2). The dihedral angles between the benzene rings in Cg′–Cg″ are 1.95° and 0.86° in the LTP and RTP structures, respectively (electronic supplementary material, figure S4). These results suggest that the 3-nitroanilinium cations move slightly along the C–N bond in the space between the supramolecular cations A and B.

**Table 2.** Dihedral angles and average Cg′ and Cg″ distances with *π*–*π* interactions along the *a*-axis at 100 and 296 K.

angle (°)	∠Cg′–Cg″	∠Cg″–Cg″	∠Cg″–Cg′	∠Cg′–Cg′
100 K	1.95	0.00	1.95	0.00
296 K	0.86	0.00	0.86	0.00

distance (Å)	d(Cg′–Cg″)	d(Cg″–Cg″)	d(Cg″–Cg′)	d(Cg′–Cg′)
100 K	3.484	3.580	3.484	3.544
296 K	3.663	3.627	3.552	3.609

**Figure 5.**
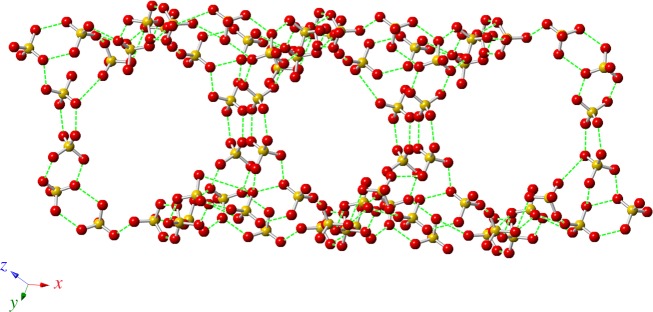
View of the 3D framework packing of the H_2_PO_4_^−^ anions and H_3_PO_4_ and H_2_O molecules by O–H···O hydrogen-bonding interactions. The dashed lines indicate hydrogen bonds.

**Figure 6.**
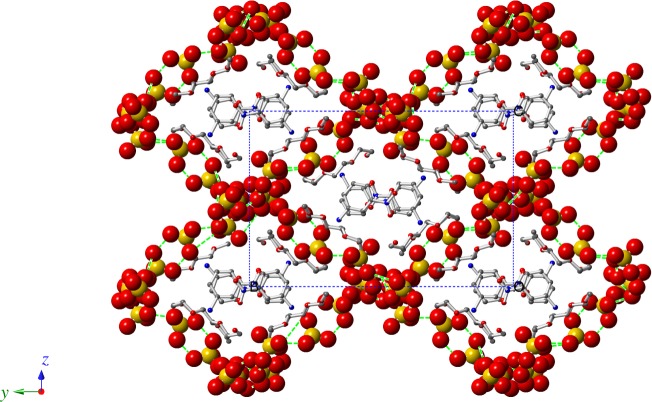
Crystal packing of **1** along the *a*-axis. The independent supramolecular cations are located in the 1D channels through *π*–*π* intermolecular interactions and insert into the cavity of the 3D framework by O–H···O hydrogen bonds.

**Figure 7.**
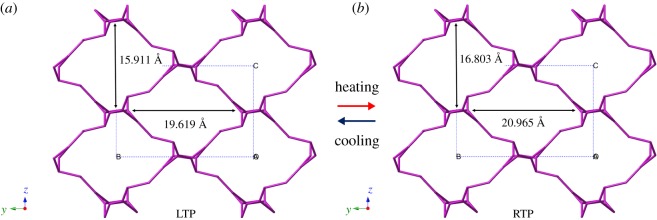
Schematic model of the 3D framework at (*a*) 100 K (LTP) and (*b*) 296 K (RTP). The solid lines indicate the conformational changes viewed along the *b-* and *c-*axes.

According to the law of charge conservation, the inorganic anions in the non-supramolecular cation part of **1** consist of two H_2_PO_4_^−^ (dihydrogen phosphate) anions, three H_3_PO_4_ (neutral phosphoric acid) molecules and one H_2_O molecule. In the LTP and RTP structures, O–H···O hydrogen bonds were formed by two H_2_PO_4_^−^ anions, three H_3_PO_4_ molecules and one H_2_O molecule, resulting in the formation of a three-dimensional (3D) framework with one-dimensional (1D) channels (figure 5). The average O···O bond lengths are 2.558 Å (LTP) and 2.670 Å (RTP), and the O–H···O bond lengths fall in the range of 2.436–2.839 Å (LTP) and 2.519–3.169 Å (RTP). The large deviation of the O–H···O hydrogen bond distance in the three-dimensional frameworks displays a much shorter average hydrogen bond distance in the LTP structure than in the RTP structure. The change of the relative position of the H_2_PO_4_^−^ anion, H_3_PO_4_ molecules and H_2_O molecules leads to a 3D deformation of the anionic framework on going from the LTP structure to the RTP one, causing the protons to move more easily within the O–H···O hydrogen-bonding network. In the crystal packing structure, independent supramolecular cations are located in the 1D channels through *π*–*π* intermolecular interactions and insert into the cavities of the 3D framework by O–H···O hydrogen bonds (figure 6; electronic supplementary material, figures S7 and S8). As shown in figure 7, we measured the size of the cavity in the direction of the *b*-axis; it is 20.965 Å in the RTP structure, which is slightly longer than that in the LTP structure (19.619 Å). Meanwhile, the cavity sizes in the direction of the equatorial plane are 15.911 Å in the LTP structure and 16.803 Å in the RTP structure. Hence, the relatively small cavity reduces the extent of pendulum-like rotational motion of the nitro group of the 3-nitroanilinium cation.

### 3.4. Potential energy calculations

**Figure 8.**
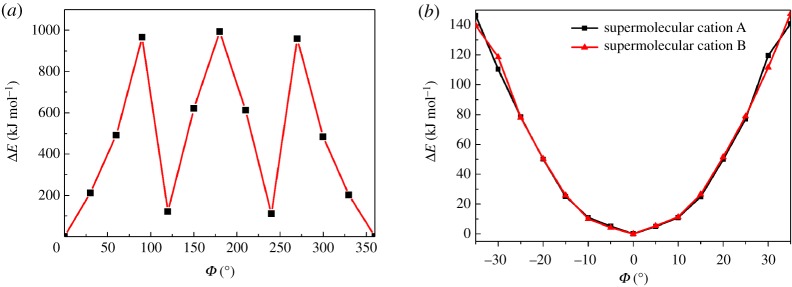
Potential energy curves for (*a*) rotational motion of the phenyl ring of the 3-nitroanilinium cation along its C–N bond and (*b*) pendulum-like motion of the nitro group in the range of −35° to 35°.

To confirm whether the motions of the 3-nitroanilinium cations and nitro groups make significant contributions to the phase transitions, the potential energies for (i) rotation of the phenyl ring and (ii) pendulum-like motion of the nitro group were calculated with the RHF/6-31(d) level of theory. These calculations were based on the fixed atomic coordinates obtained from the crystal structure at 296 K. The model used for these calculations is shown in electronic supplementary material, figure S9a. For the phenyl ring motion along the C_25_–N_1_ axis, the atomic coordinates of the −NH_3_^+^ groups were kept fixed while rigid motion of the phenyl ring was applied to calculate the rotational potential energy. Three 18-crown-6 molecules, two 3-nitroanilinium cations, and two H_2_PO_4_^−^ anions were included in the models to evaluate the effects of steric hindrance. The rotational angle (*Ф*) dependencies of the potential energies (Δ*E*) for the phenyl ring in compound **1** from 0° to 360° at 30° increments are provided in figure 8*a*. The initial atomic coordinates obtained from the crystal structure at 296 K correspond to the first potential energy minimum at *Ф* = 0°, whose relative energy was defined as zero. The second and third potential energy minima appear at *Ф* = 120° and 240°, respectively, for the phenyl ring in **1**, and the maxima were calculated at *Ф* = 90°, 180° and 270°, with corresponding Δ*E* values of 965.90, 993.05 and 958.24 kJ mol^−1^, respectively. The magnitude of the maximum Δ*E* for the phenyl ring (≈1000 kJ mol^−1^) was far greater than that in (m-fluoroanilinium^+^)(DB[18]-crown-6)[Ni(dmit)_2_]^−^(≈270 kJ mol^−1^) [8]. The high potential energy barriers for rotation of the phenyl ring suggest that it is almost impossible above room temperature.

The pendulum-like motion of the nitro group was evaluated with fixed atomic coordinates of the C_6_H_4_–NO_2_ moiety in the supramolecular cations. The models used for these calculations comprise three supramolecular cations [(3-nitroanilinium)(18-crown-6)]^+^ (electronic supplementary material, figure S9b and S9c). Because rotation of the nitro group by 360° is very likely to be impossible because of the large steric hindrance of the neighbouring phenyl rings, the relative energy of pendulum-like motion of the nitro group was calculated at 5° increments within the range of −35° to 35°. Two symmetrical potential energy profiles were calculated for this motion in the supramolecular cations A and B (figure 8*b*). The magnitude of the maximum Δ*E* was *ca* 140 kJ mol^−1^ (less than 270 kJ mol^−1^), indicating that pendulum-like motion of the nitro group around the C_27_–N_2_ and C_33_–N_4_ bonds is possible at 296 K. Consequently, such motion is likely to be restricted at 100 K but easily activated as the temperature is increased. Overall, these results suggest that the pendulum-like motion of the nitro group may well be related to the observed phase transitions.

### 3.5. Dielectric properties

**Figure 9.**
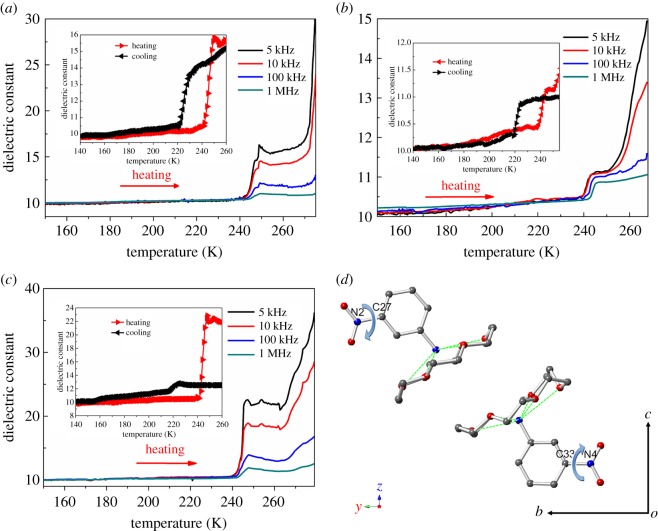
Anisotropic dielectric constants of **1** along (*a*) *a-*, (*b*) *b-* and (*c*) *c-*axes at 5 kHz to 1 MHz upon heating. (*d*) The pendulum-like motion of the nitro-group in supramolecular cations A and B corresponds to the direction of the *ac*-plane. Insert: the thermal hysteresis curves at 5 kHz.

It is well known that physical properties usually display abrupt changes in the vicinity of a phase transition, and the magnitude is correlated to characteristic features of the phase transition. The dielectric properties of **1** were measured in three directions of a single-crystal sample. The temperature dependence of the real parts (*ɛ′*) of the dielectric constants taken at 5 kHz, 10 kHz, 100 kHz and 1 MHz are depicted in figure 9 and electronic supplementary material, figure S10. The *T*–*ɛ′* curve shows two obvious step-like dielectric anomalies in the temperature range of 150–280 K, which is consistent with the results determined using DSC. At 5 kHz, *ɛ′* remains at approximately 11–12 from 150 to 242 K; it then increases to a maximum of approximately 17 at *ca* 250 K along the *a*-axis (figure 9*a*). Figure 9*b* and electronic supplementary material, figure S10b display two clear step-like dielectric anomalies at approximately 225 K on cooling and 242 K on heating. For the *c*-axis, two step-like dielectric anomalies similar to that observed for the *a-* and *b-*axes were also observed (figure 9*c* and electronic supplementary material, figure S10c). In the heating mode, *ɛ′* remains stable (approx. 10) until the temperature reaches *ca* 242 K at different frequencies, corresponding to the low dielectric state. With rising temperature, the *ɛ′* exhibited a prominent increase from *ca* 10 to 25, 20, 14, and 12 at 5 kHz, 10 kHz, 100 kHz, and 1 MHz, respectively. Upon cooling again, the curves plateau with a slight decrease and the *ɛ*′ values change from *ca* 12.8 to 11.8 at a frequency of 5 kHz. The changes of *ɛ′* at lower frequencies are more pronounced than those at higher frequencies, indicating that the dielectric constant is very sensitive to external frequencies. From analysis of the crystal structure of **1**, the dielectric anisotropy was related to the anisotropy of proton transfer in the 3D framework through O–H···O hydrogen bonds. In addition, the temperature-dependent curves of *ɛ′* along the *a-*, *b-* and *c-*axes obtained in the cooling mode match well with those obtained during the heating process, suggesting the occurrence of a reversible phase transition.

Distinct step-like anomalies around 241 K can be viewed along both the *a-* and *c-*axes, while relatively smaller anomalies are apparent along the *b*-axis. These phenomena can be explained by examining the dynamic motion of the nitro group in the cation. Pendulum-like motion of this group occurs in the *ac*-plane, which enhances the *ɛ′* along the *a-* and *c-*axes. The fluxional frequency of the nitro group motion coupling with proton transfer is suppressed as the temperature decreases, when they become frozen in a given state. Therefore, there is no obvious change in the value of *ɛ′* with changing frequency; it does increase with increasing temperature, however. Further dielectric enhancement was observed at temperatures above 260 K, where the low-frequency and *ɛ′* was larger than the high-frequency values. Importantly, the phase transition of compound **1**, which can be tuned between low and high dielectric states at low and room temperature, makes it an excellent candidate for molecular switchable dielectrics.

## 4. Conclusion

In conclusion, a new three-dimensional organic–inorganic hybrid compound, (3-nitroanilinium)_2_(18-crown-6)_2_(H_2_PO_4_)_2_(H_3_PO_4_)_3_(H_2_O) (**1**), was synthesized. Compound **1** undergoes a reversible phase transition at *ca* 231 K, which is anisotropy in the vicinity of *T*c. The dynamic behaviours between the pendulum-like motions of the nitro group in the organic cations and proton transfer in the O–H···O hydrogen bonds of the 3D framework produce a synergistic effect that gives rise to the observed phase transition and distinct dielectric anisotropy along various crystallographic axes. Notably, the dielectric constants could be switched by continuous phase transitions and tuned in the low and high dielectric states, indicating the potential application of structures like **1** in switchable dielectrics. We believe that this example will inspire new strategies for the successful design of other organic–inorganic hybrid multifunctional materials.

## Supplementary Material

P21C.rar

## Supplementary Material

LTp-100K.cif

## Supplementary Material

supplementary material.doc

## Supplementary Material

crystal data (including checkCIF).rar

## Data accessibility

This article does not contain any additional data.
