# Supplementary material for: Phase transition and switchable dielectric behaviours in an organic–inorganic hybrid compound: (3-nitroanilinium)2(18-crown-6)2(H2PO4)2(H3PO4)3(H2O)
Source: R Soc Open Sci. 2018 Oct 3;5(10):180738. doi: 10.1098/rsos.180738 (PMC6227991; doi:10.1098/rsos.180738)
Supplement: supplementary material.doc [file rsos180738supp3.doc]

Phase transition, anisotropic, and switchable dielectric behaviours in an organic–inorganic hybrid compound: (3-nitroanilinium)_2_(18-crown-6)_2_(H_2_PO_4_)_2_(H_3_PO_4_)_3_(H_2_O)

Yang Liu, Chun-li Zhu, Xiao-yuan Zheng, Liu-lei Qin, Shuang-xi Yang and Zun-qi Liu*

*Chemical Engineering College* *Xinjiang Agricultural University,* *Urumqi 830052 China*


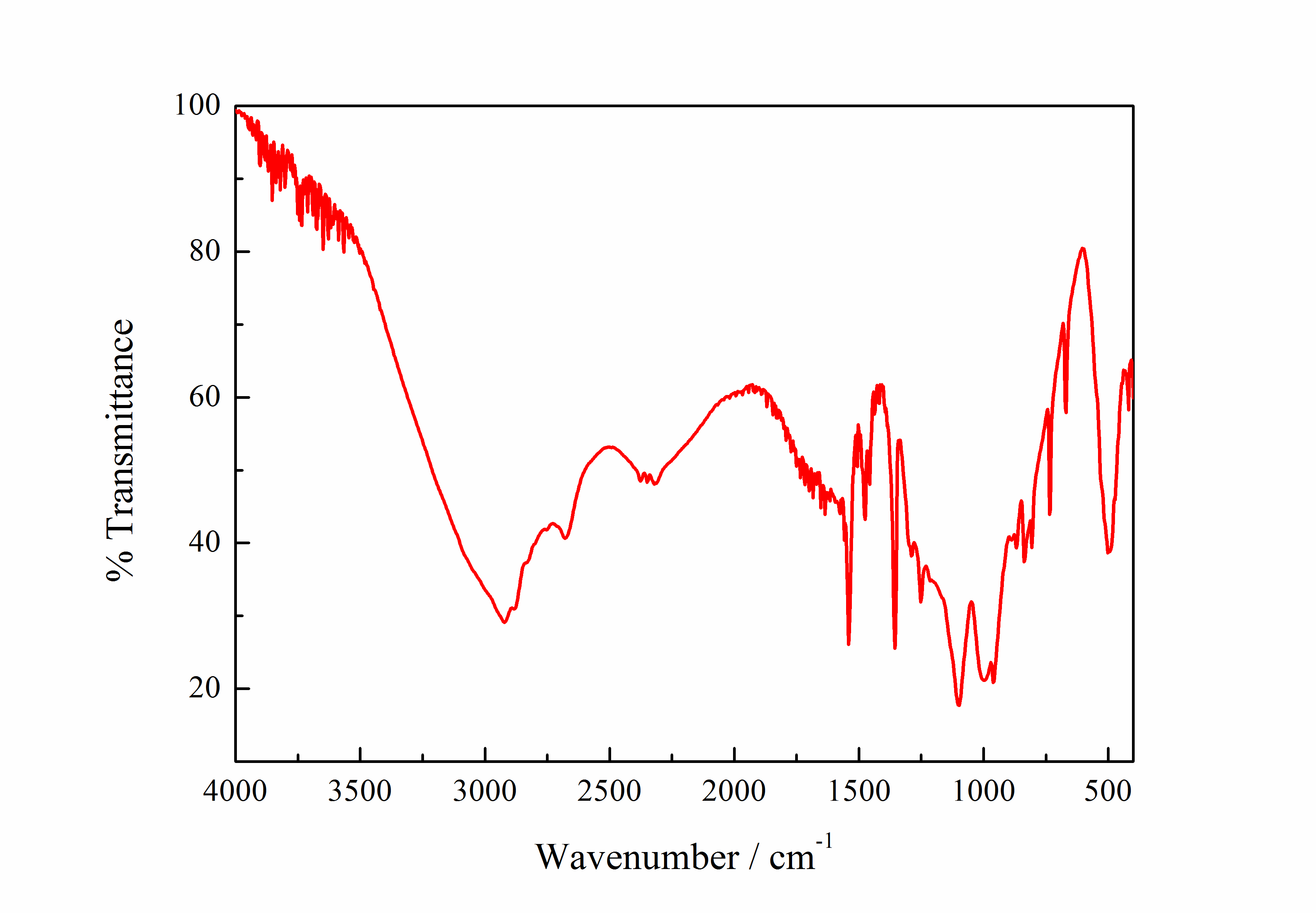


Fig. S1 IR of crystal **1**


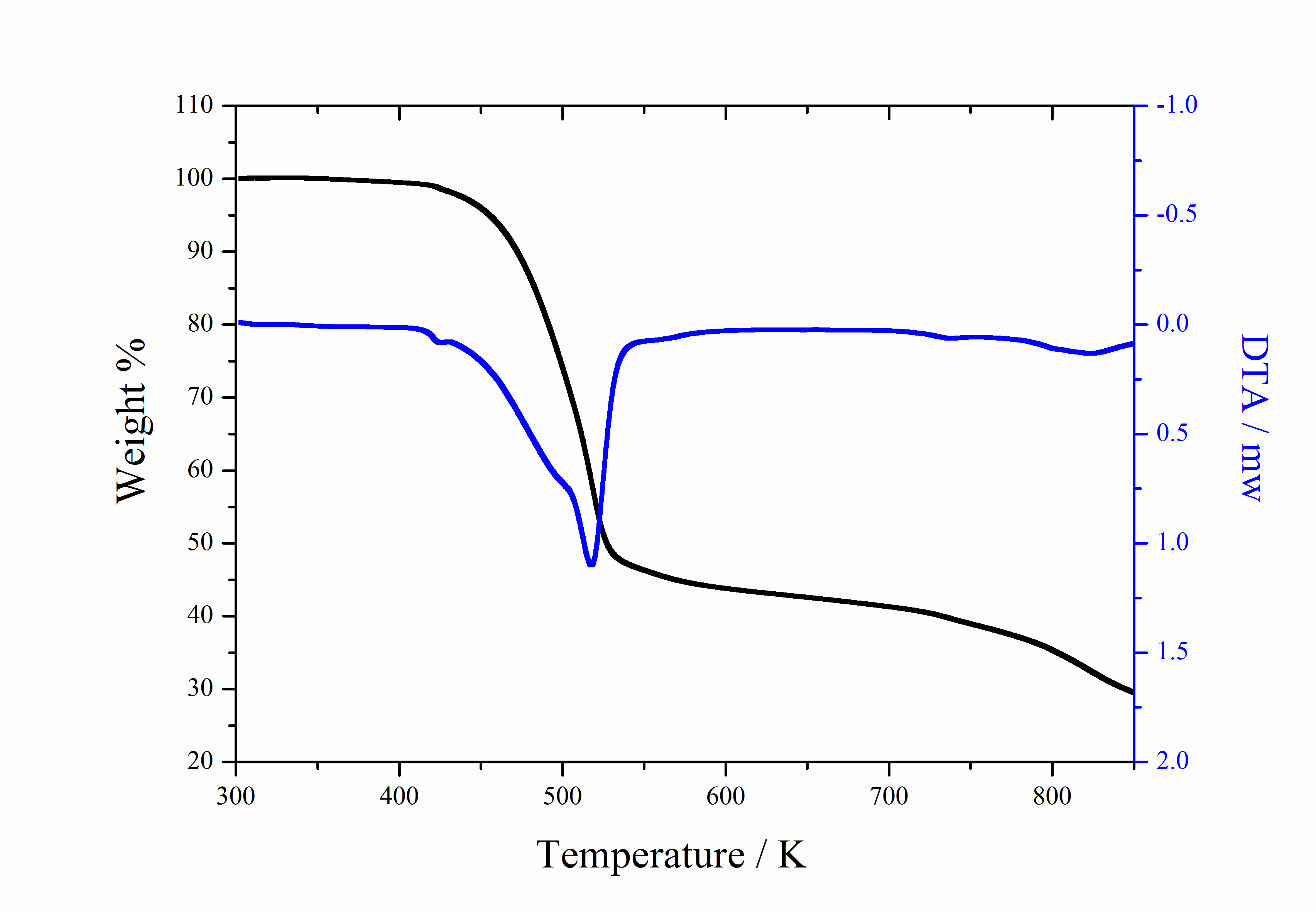


Fig. S2 TG and DTA cures for inclusion compound **1**


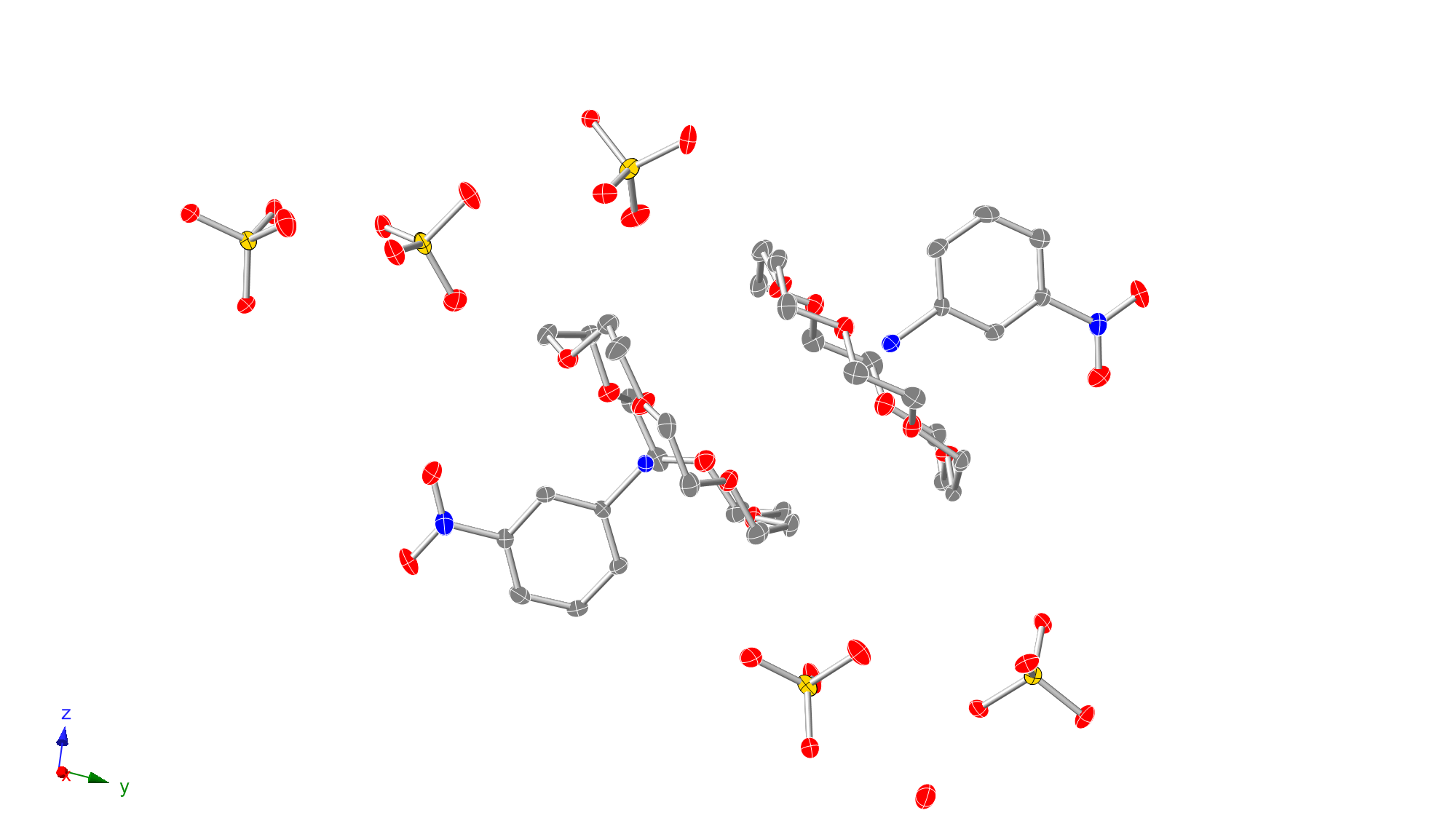


(a)


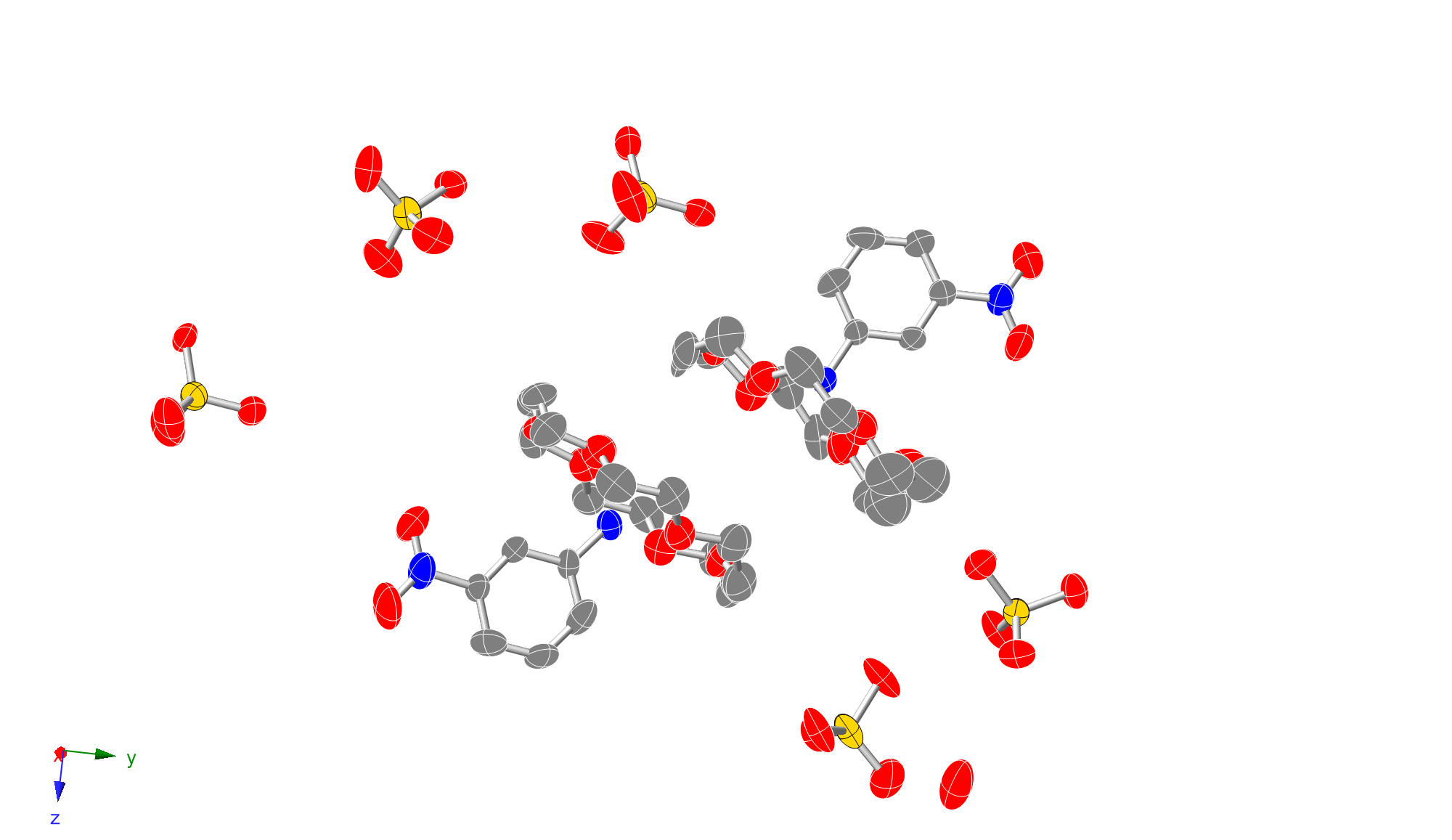


(b)

Fig. S3 View of asymmetry unit of compound **1** (a) at 100 K and (b) 296 K. Carbon-bond H atoms were omitted for clarity.


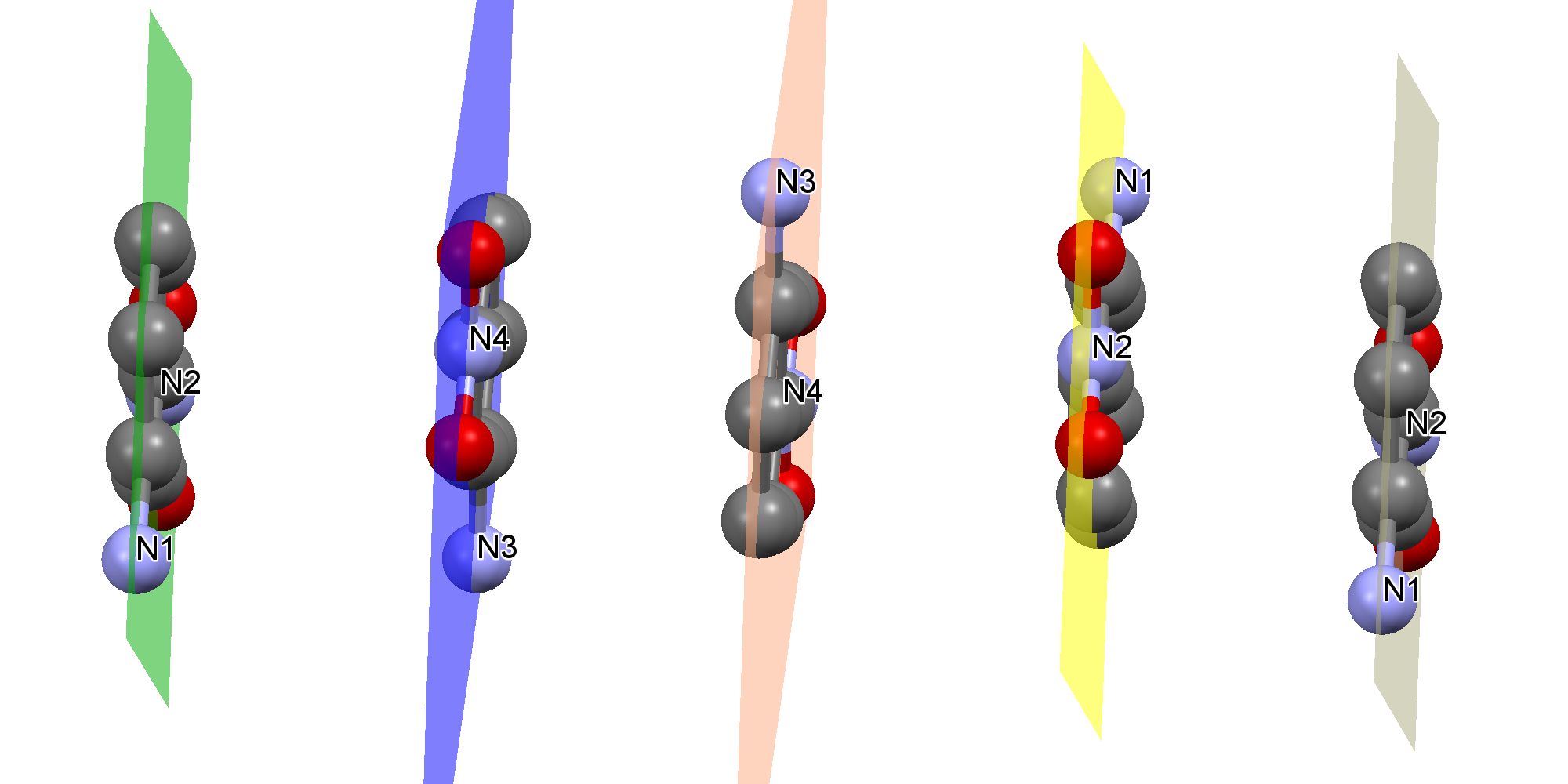


(a)


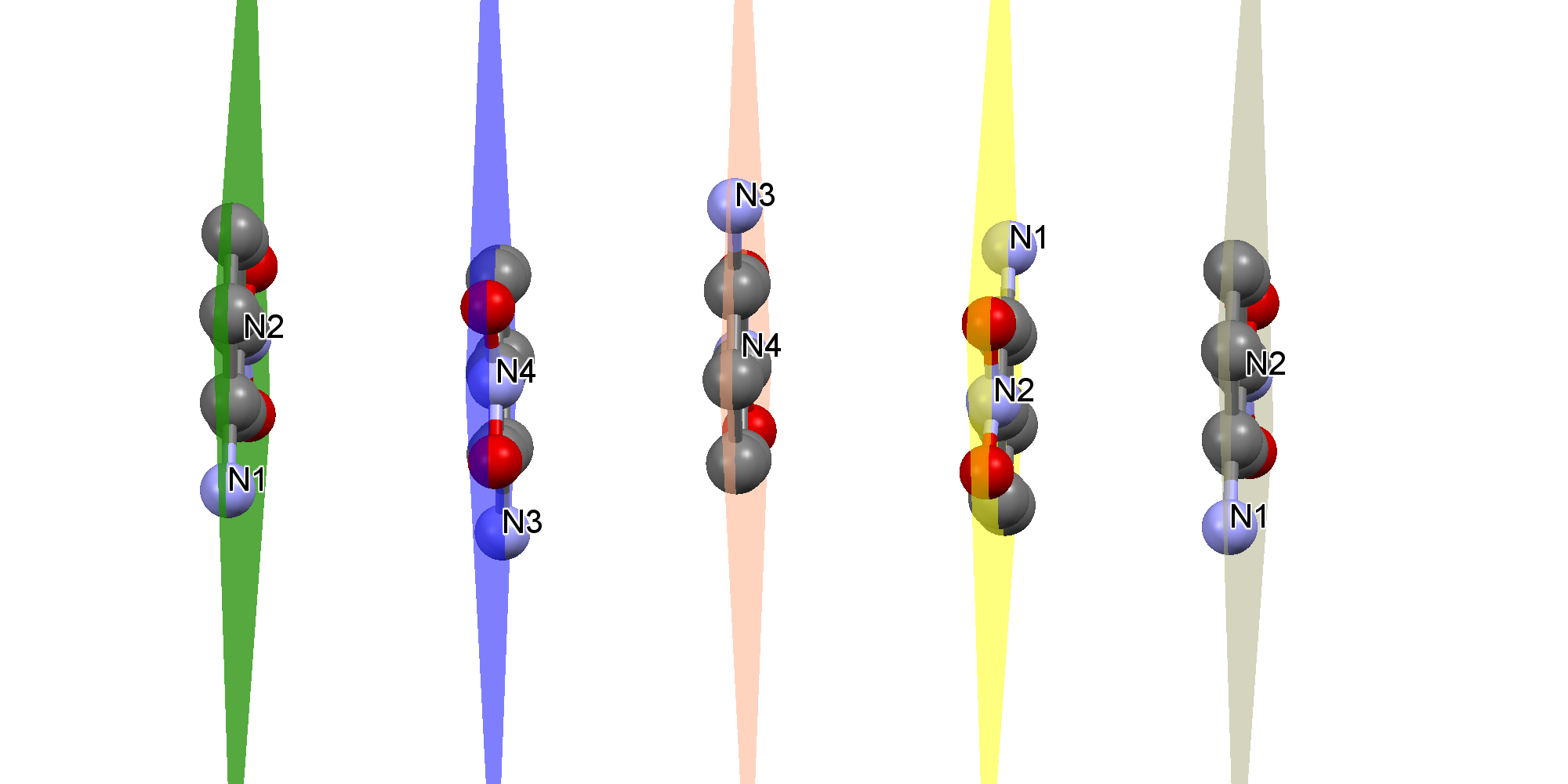


(b)

Fig. S4 One-dimensional chain structure of π-π interaction viewing down the a-axis at 100 K and 296 K, respectively, exhibit the molecular packing solvent molecules have been omitted for clarity.

Fig.S5 Dihedral angle of benzene and –NO_2_ group of supermolecular cation (3-nitroanilinium)(18-crown-6) (A) and (3-nitroanilinium)(18-crown-6) (B) in 100 K(a and b) and 296 K (c and d).


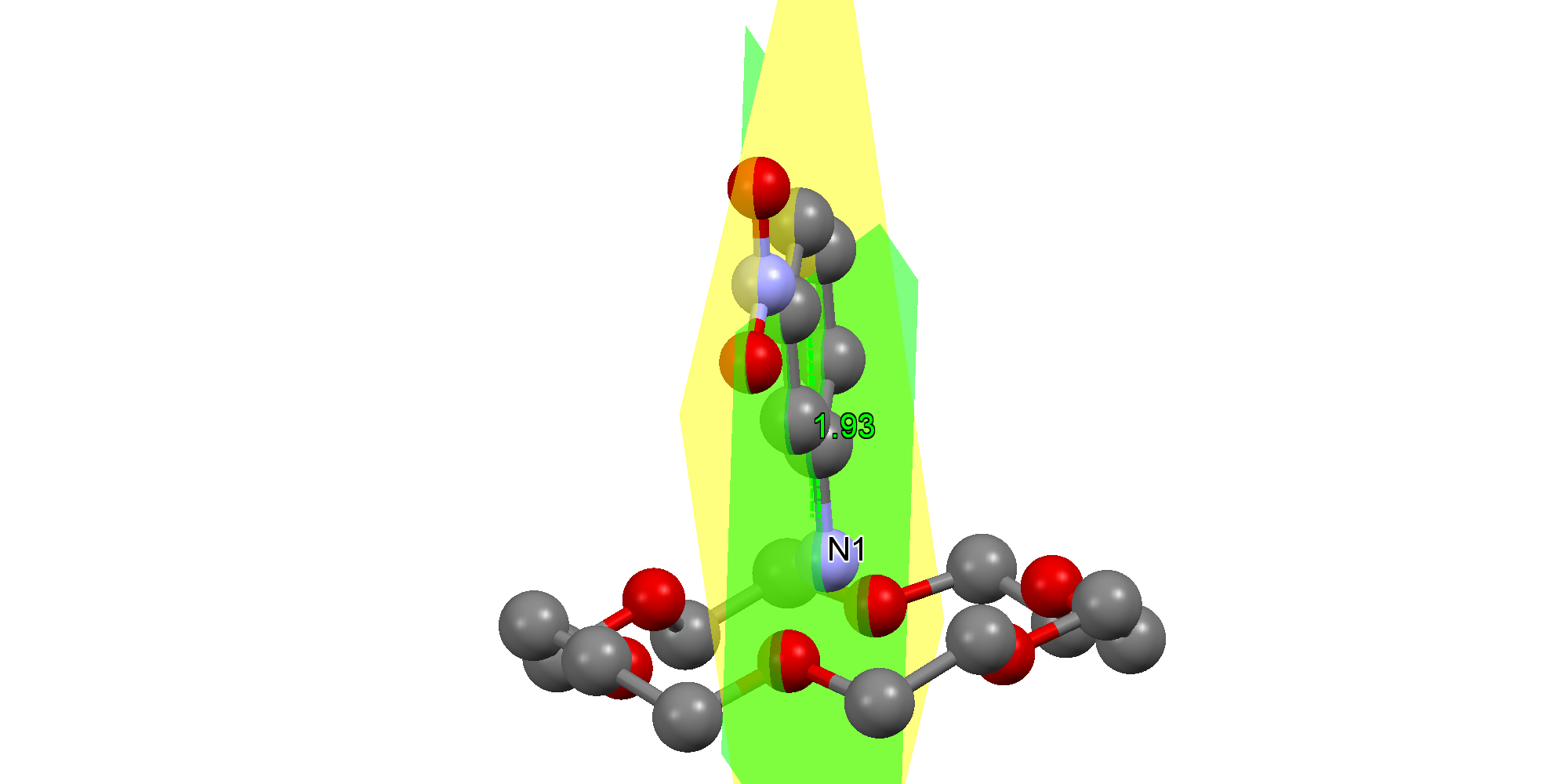


(a)

(A)


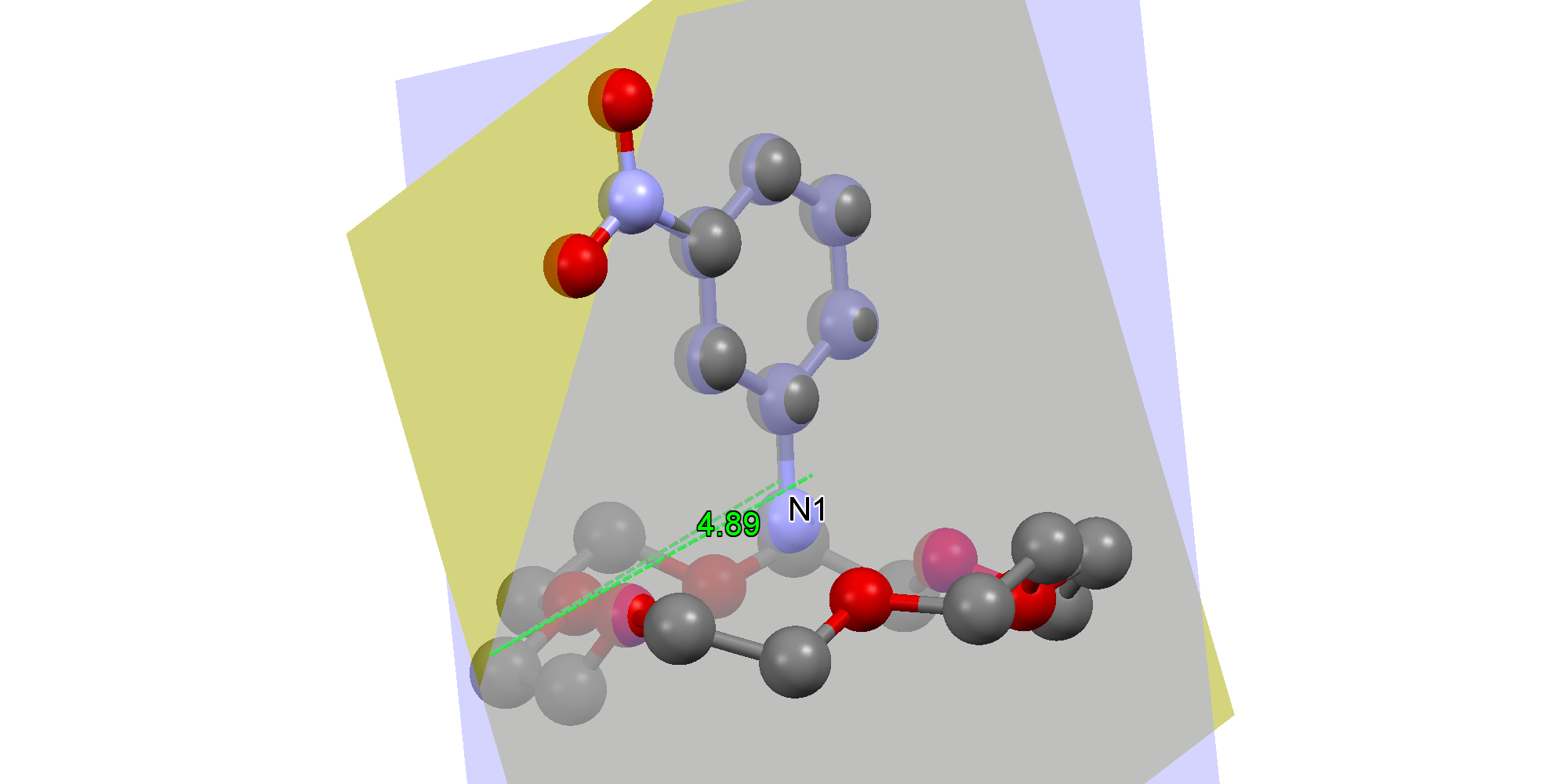


(c)

(A)


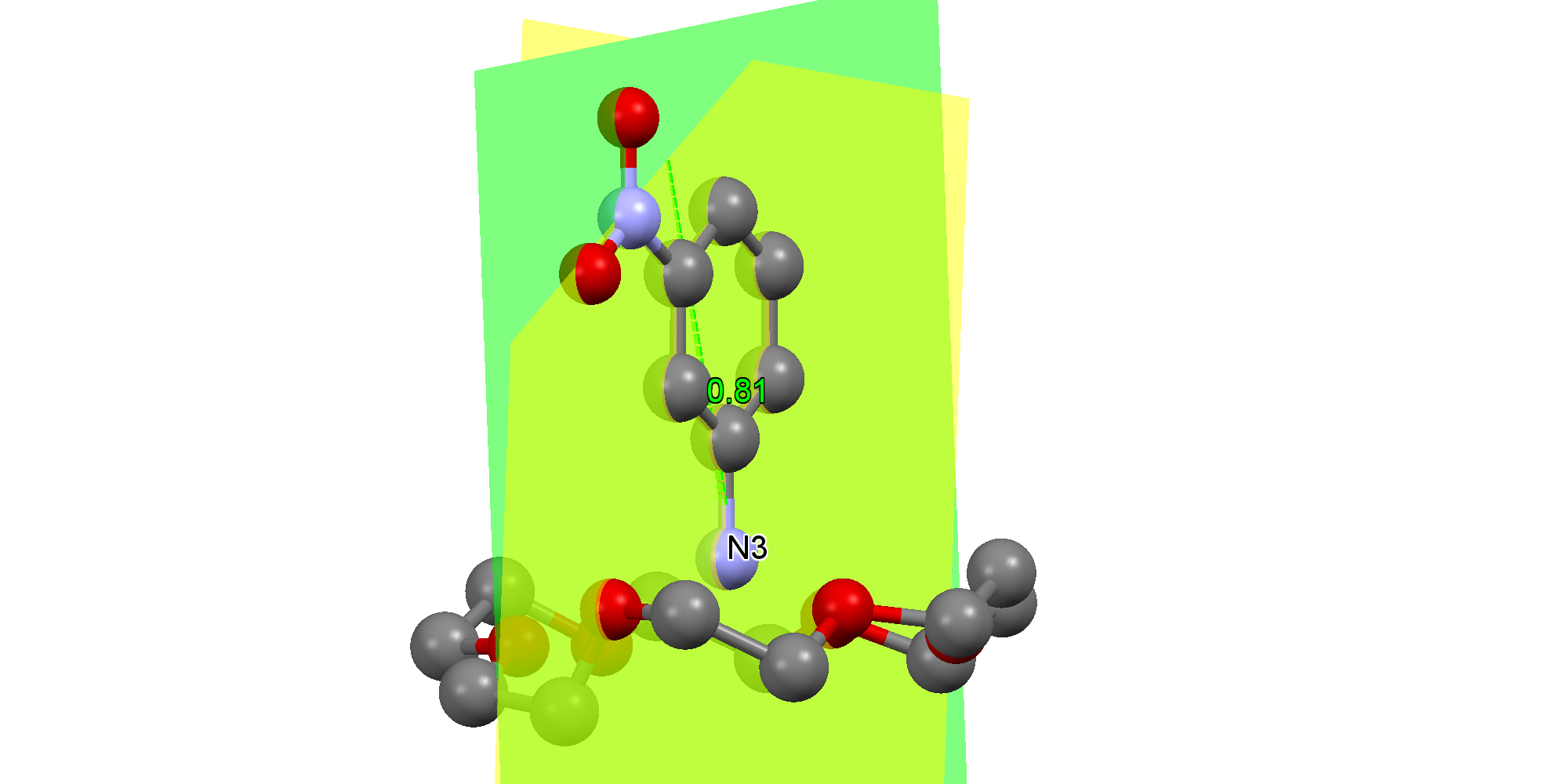


(b)

(B)


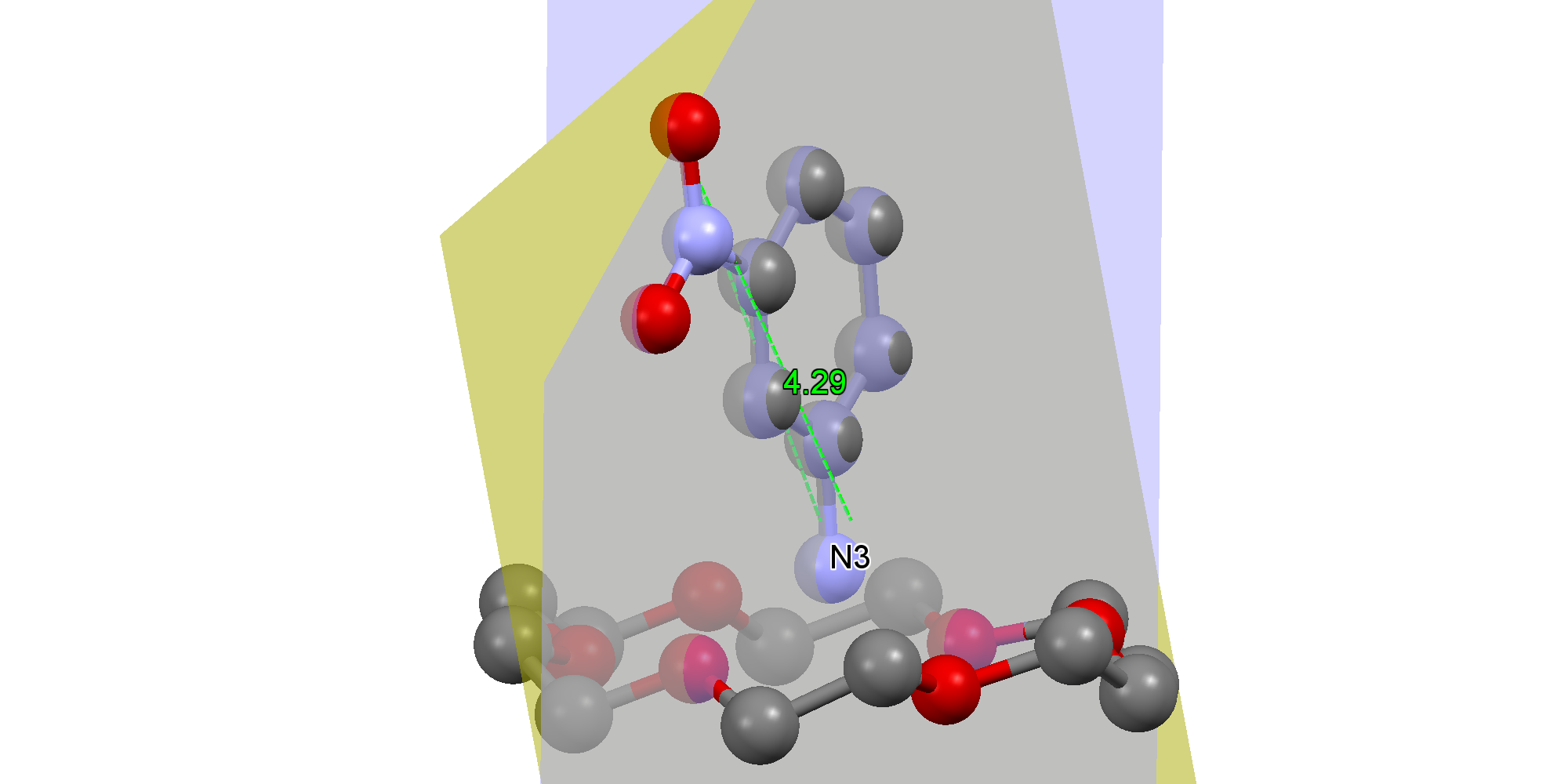


(d)

(B)


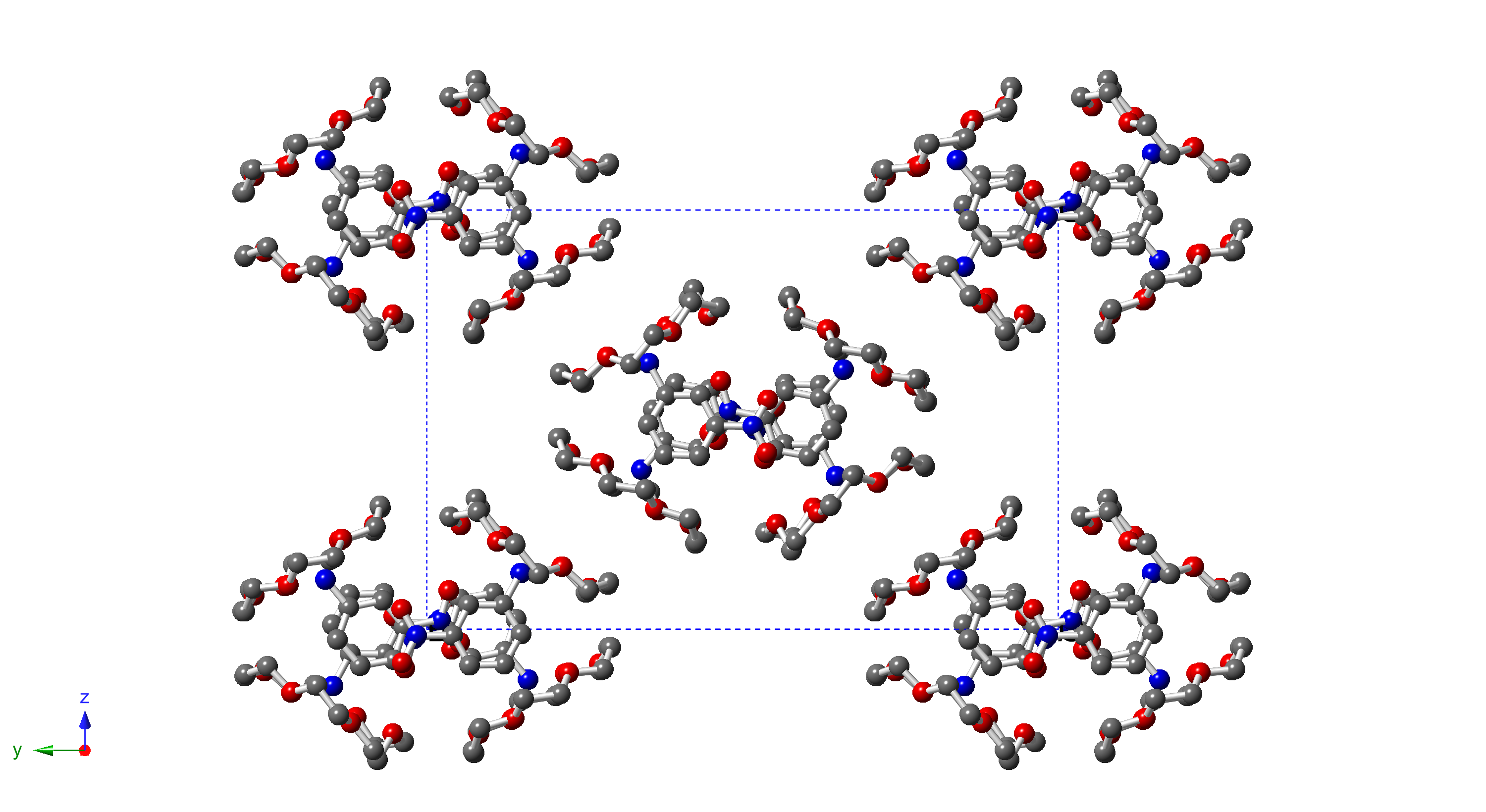


Fig. S6 One-dimensional column structure of supermolecular cation A and B through π-π interaction along a-axis. The H_2_PO_4_^-^ anion and H_3_PO_4_ molecular have been omitted for clarity.

Fig. S7 Three-dimension network schematic model of the phosphate ions with hydrogen bond O-H---O in compound **1** along *a+(b+c)*-axis


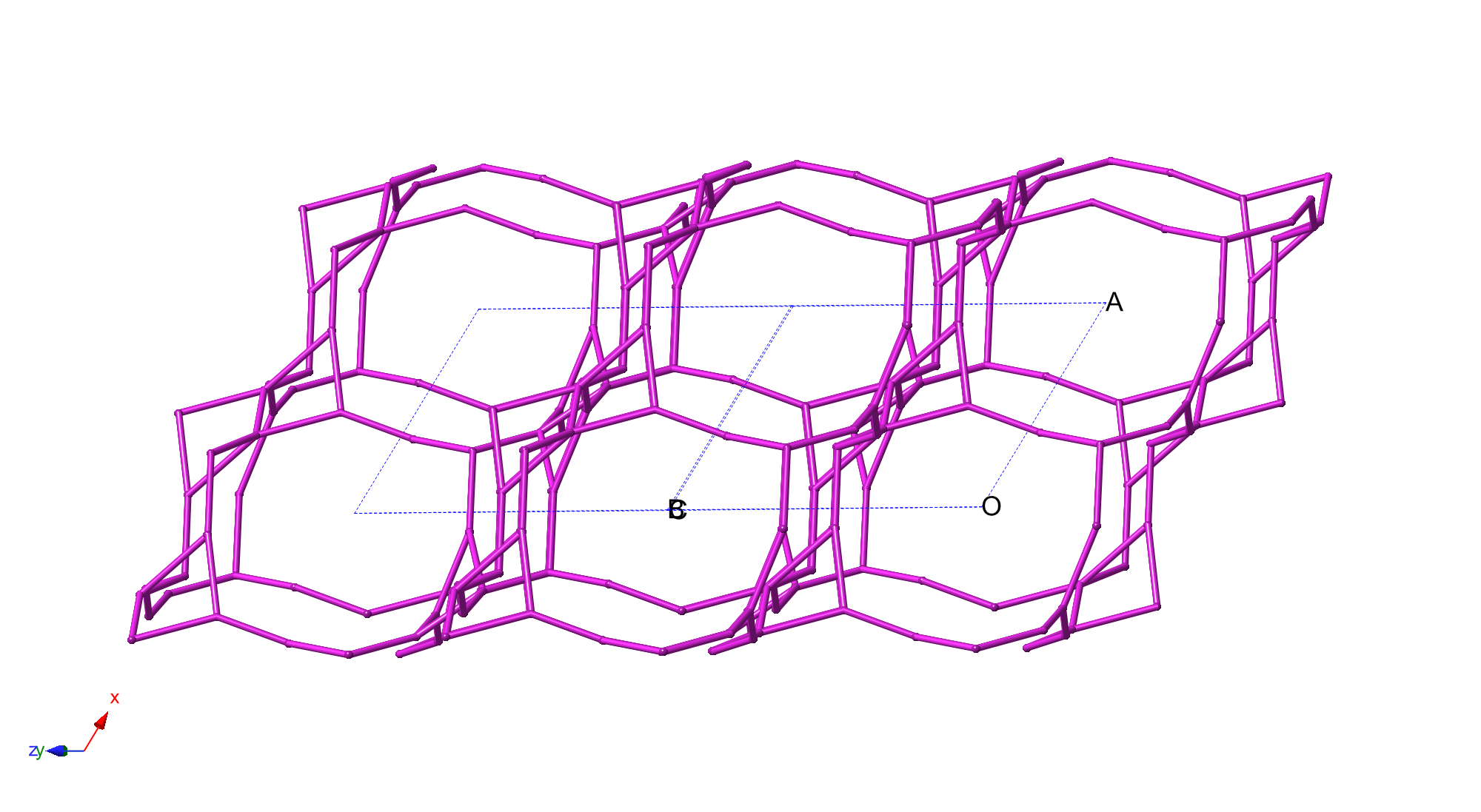

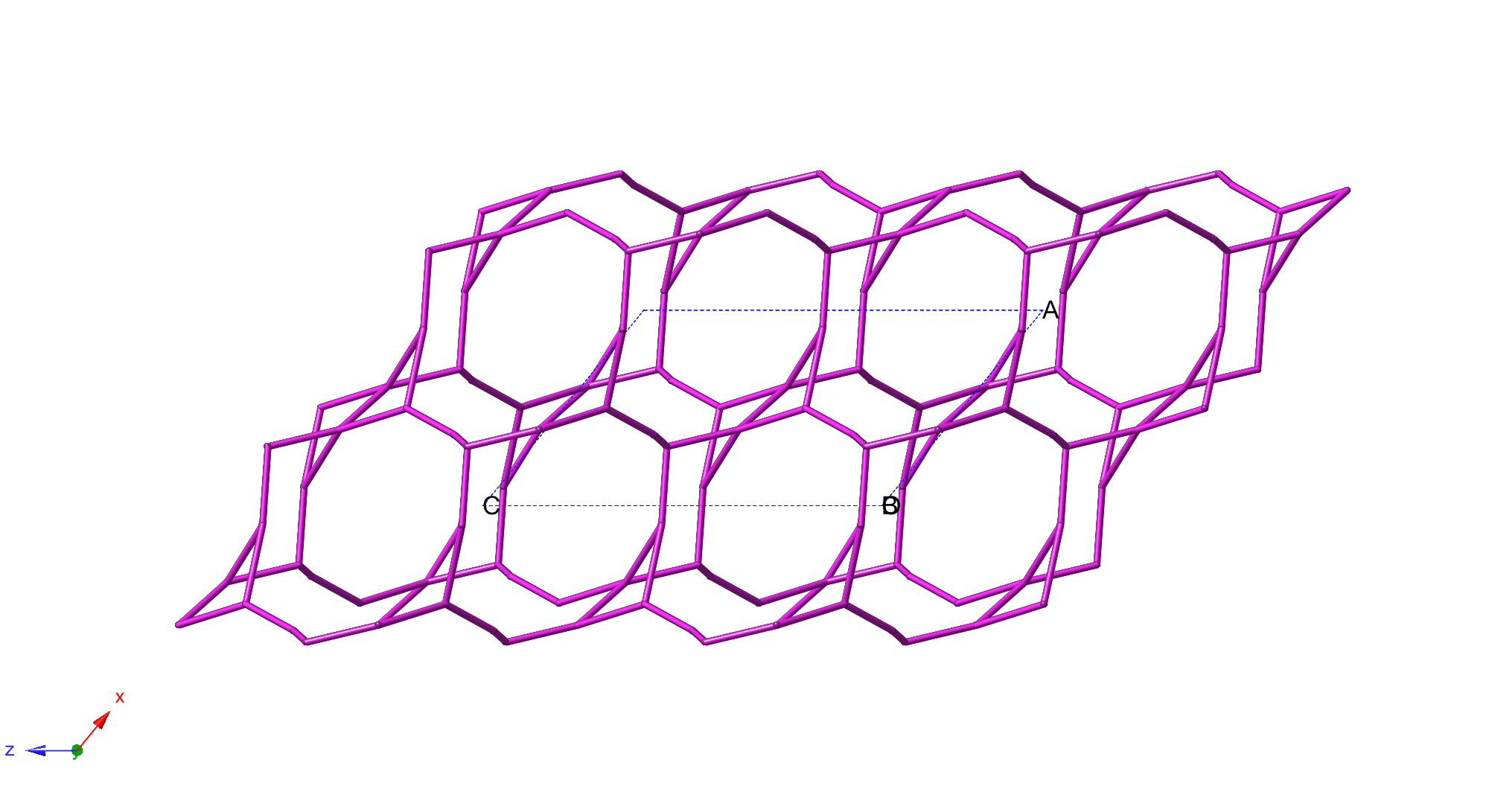


Fig. S8 Three-dimension network schematic model of the phosphate ions with hydrogen bond O-H---O in compound **1** along *b*-axis


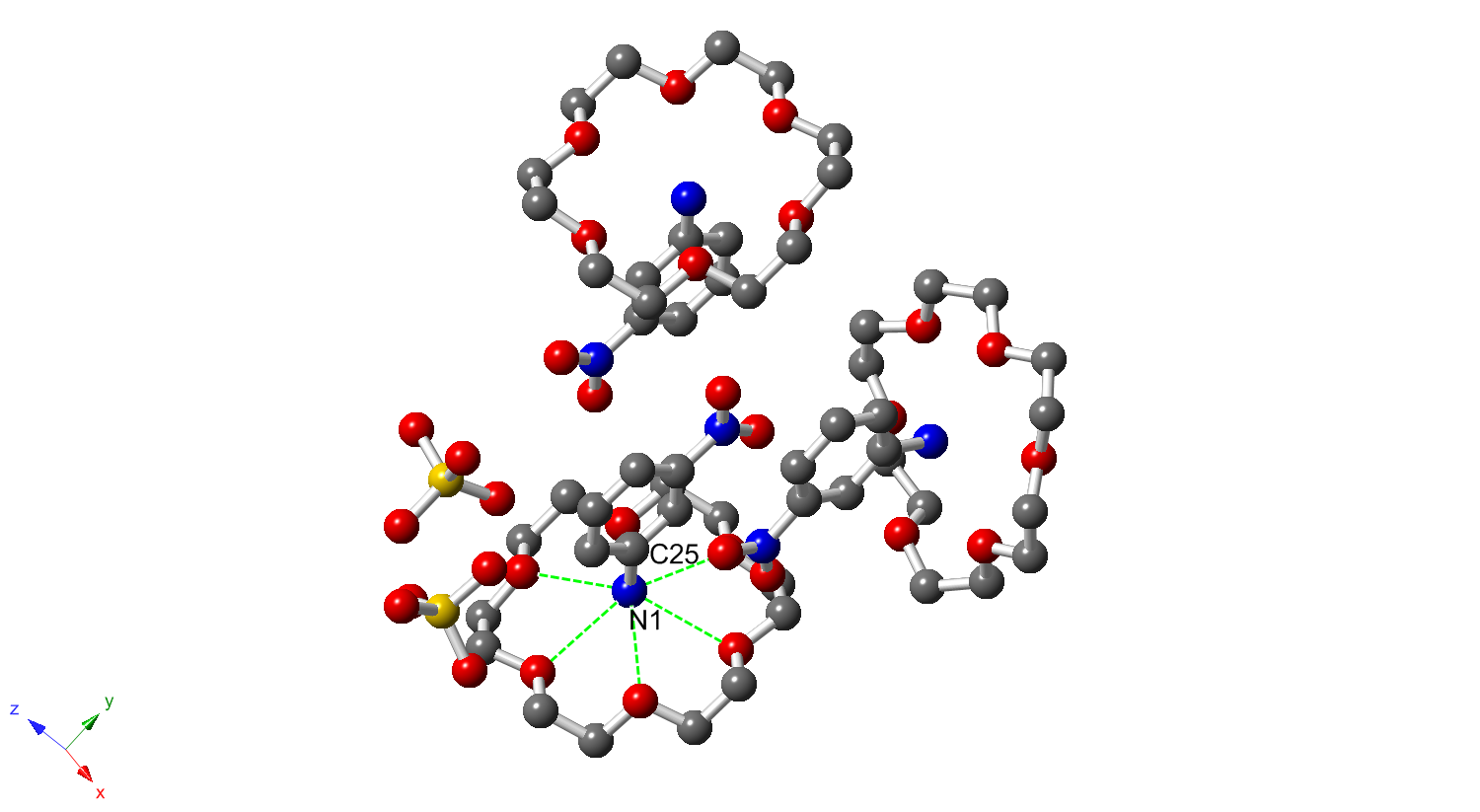


(a)


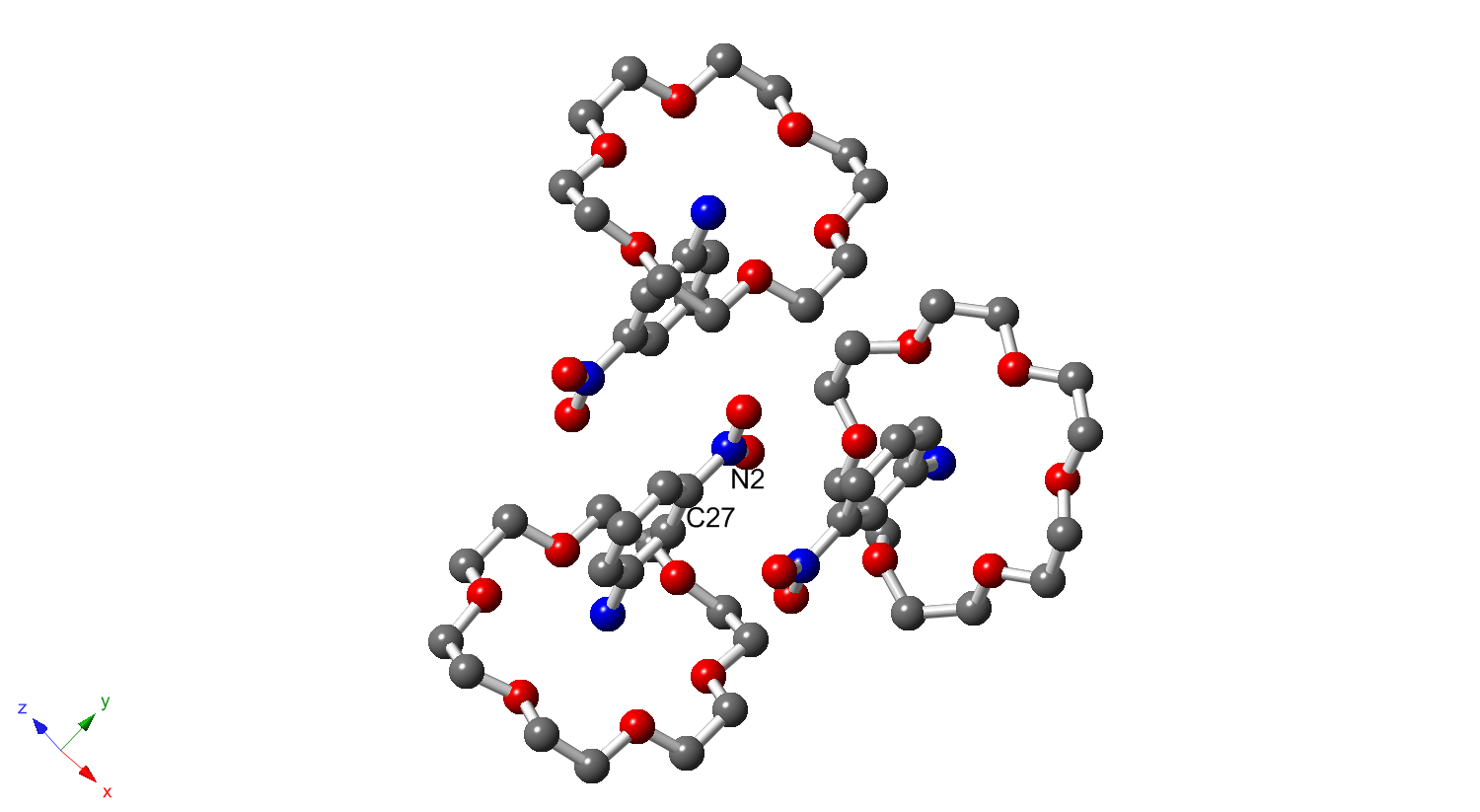


(b)


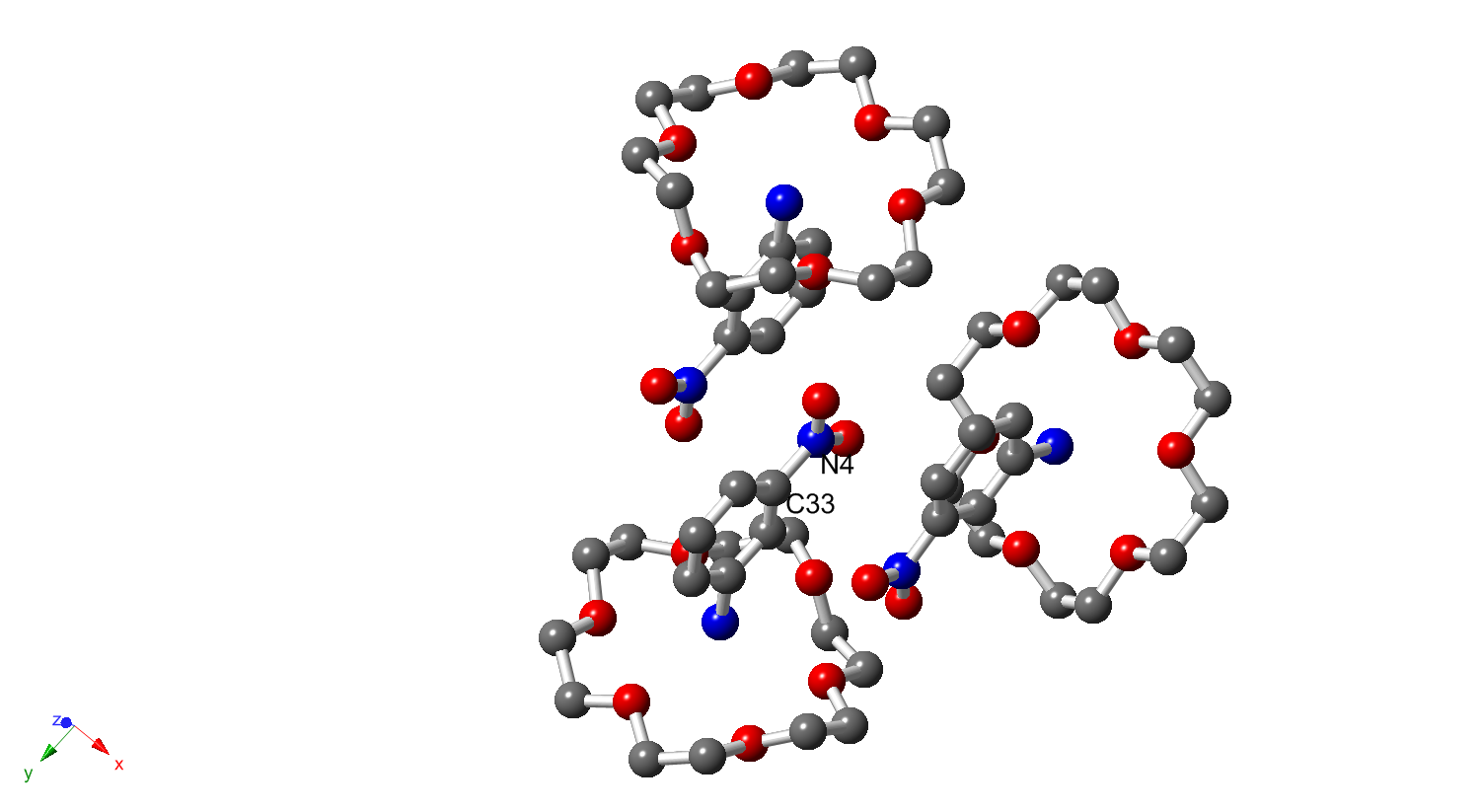


(c)

Fig. S9 Model structure of compound **1** for (a) rotation of phenyl ring, (b) pendulum motions of the nitro-group in (3-nitroanilinium)(18-crown-6) (A) and (c) pendulum motions of the nitro-group in (3-nitroanilinium)(18-crown-6) (B)


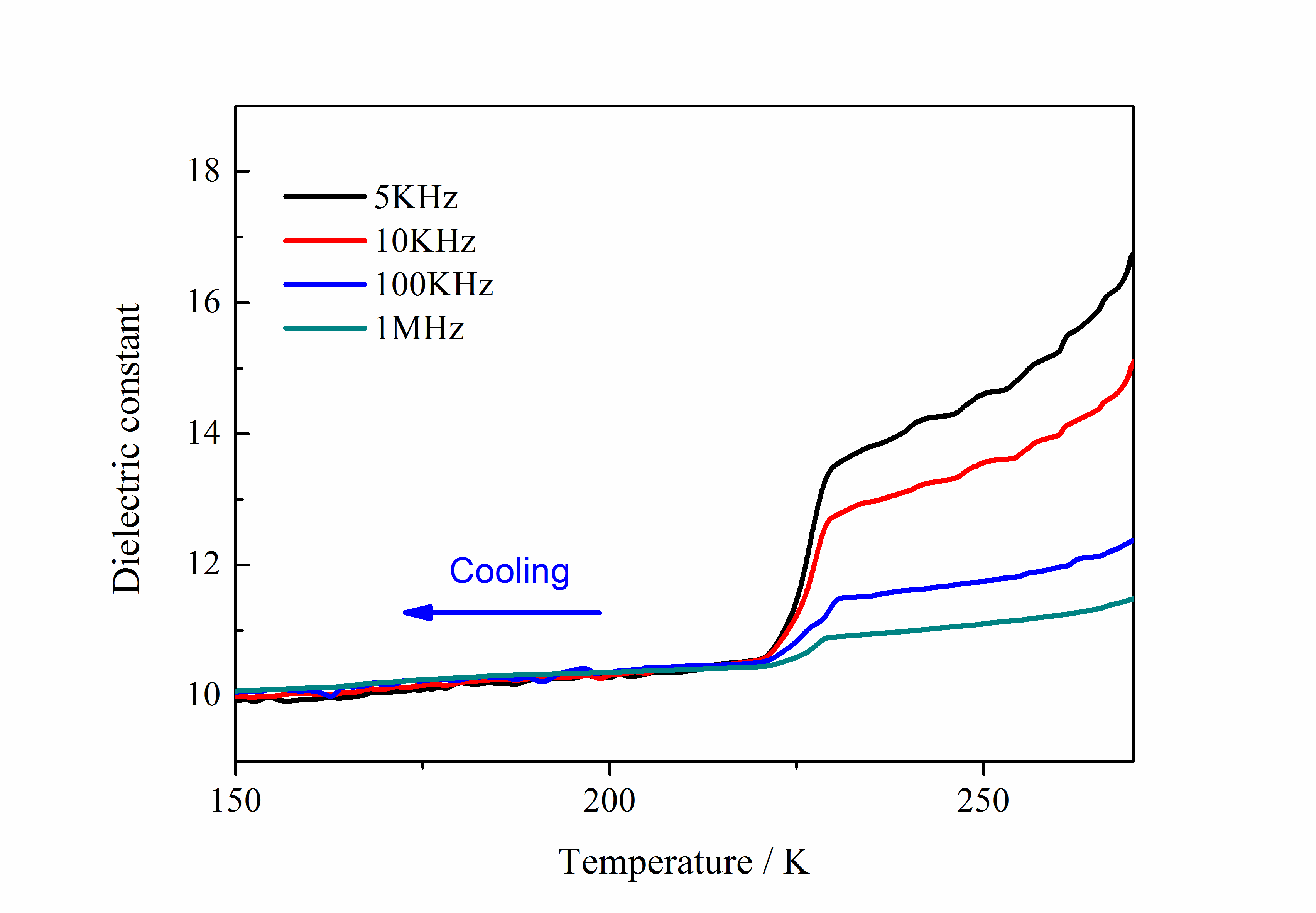


(a)


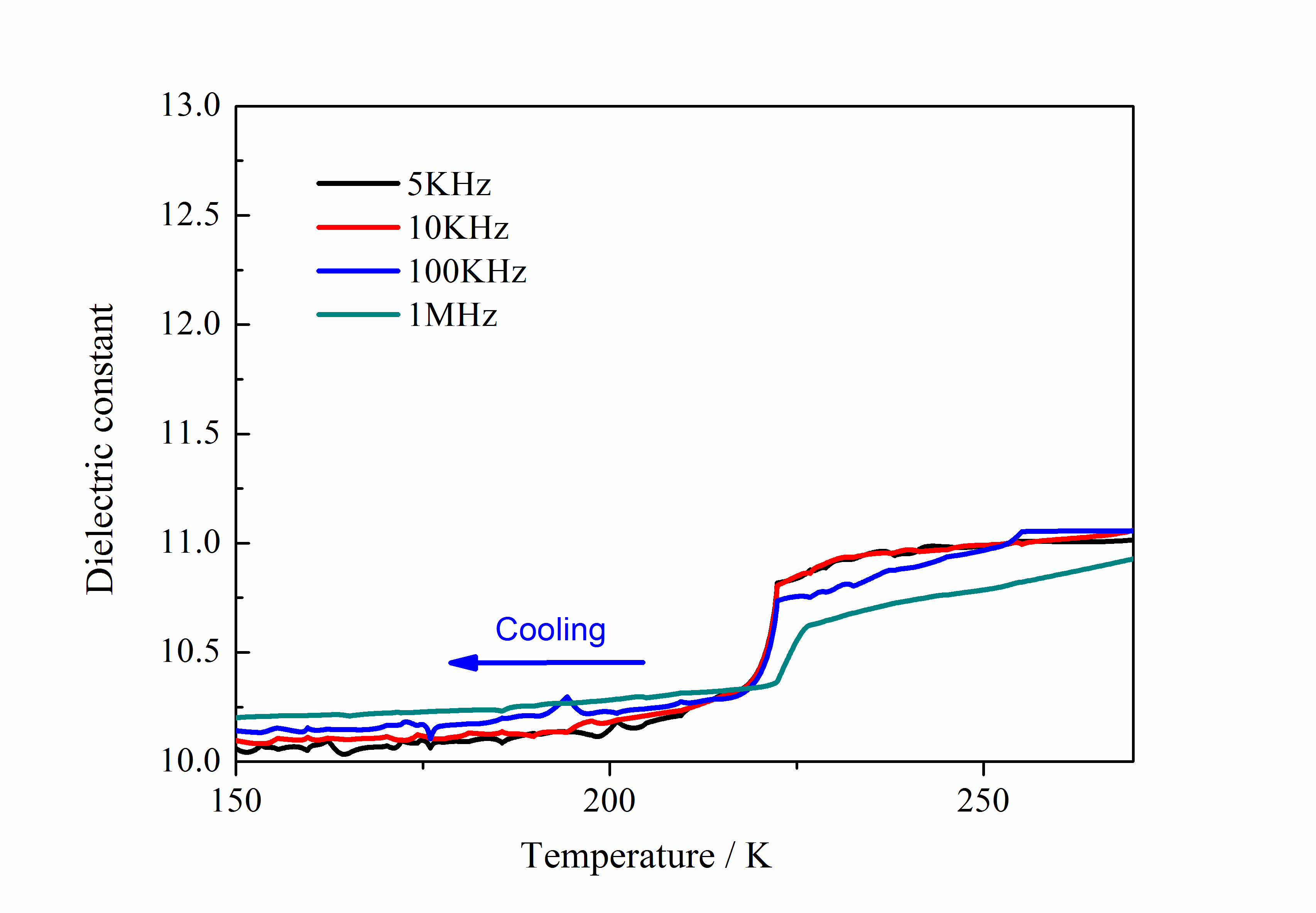


(b)


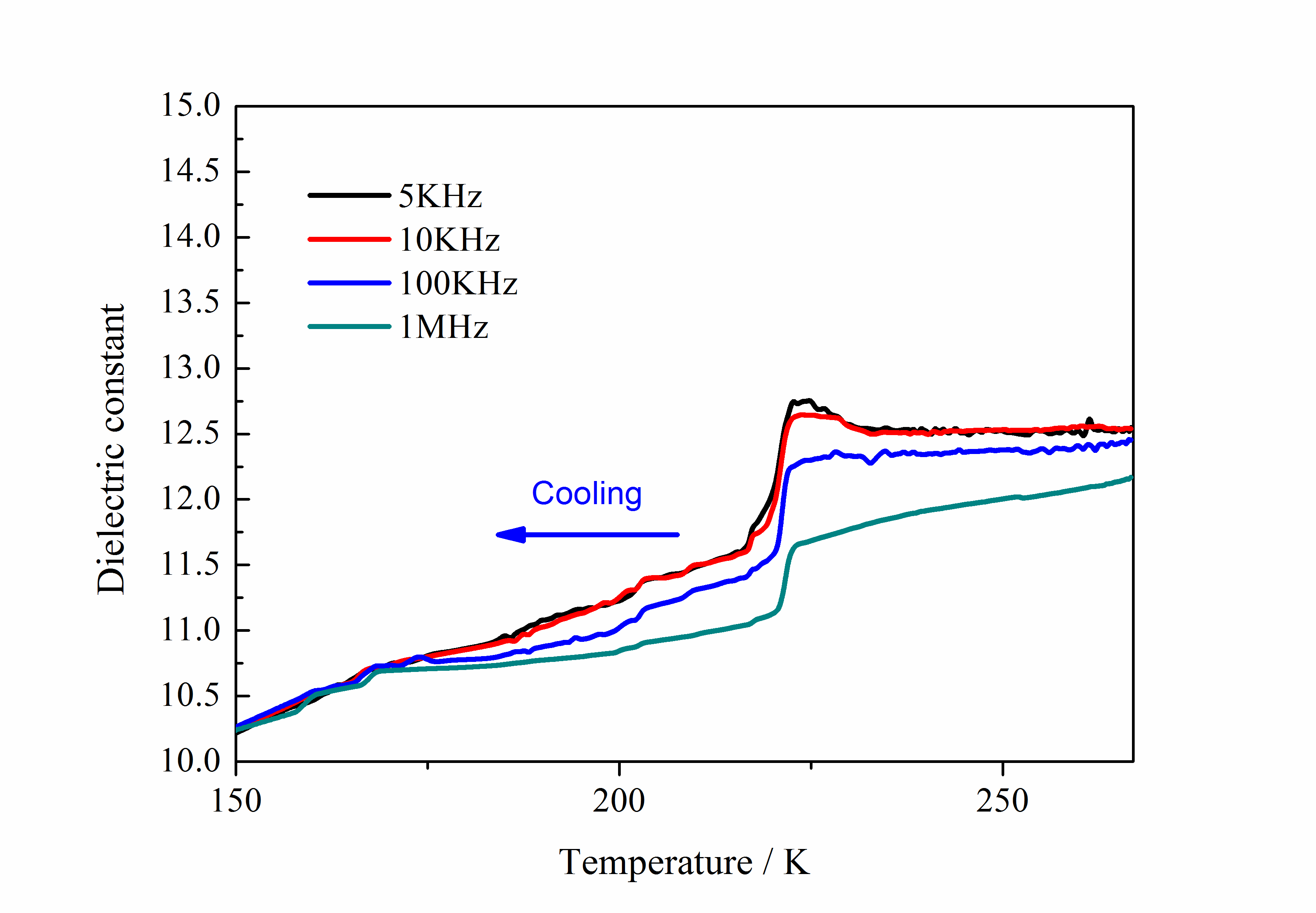


(c)

Fig. S10 Anisotropic dielectric constants of compound **1** along *a*- (a), *b*-(b) and *c*-axes (c) at 5KHz to 1MHz up cooling.

Table S1. Hydrogen-bond geometry (**Å,** deg) of supermolecule cation at 100 and 296K in compound **1**.

| D-H…A | d(D-H) **Å** | d(H…A) **Å** | d(D…A) **Å** | D-H…A(°) |
| --- | --- | --- | --- | --- |
| 100K | | | | |
| N1-H…O7 | 0.890 | 2.000 | 2.888 | 175.20 |
| N1-H…O8 | 0.890 | 2.576 | 2.929 | 104.54 |
| N1-H…O9 | 0.890 | 2.026 | 2.913 | 174.28 |
| N1-H…O10 | 0.890 | 2.534 | 2.937 | 106.46 |
| N1-H…O11 | 0.889 | 2.059 | 2.914 | 168.93 |
| N1-H…O12 | 0.890 | 2.526 | 2.916 | 107.22 |
| N3-H…O1 | 0.890 | 2.050 | 2.940 | 178.45 |
| N3-H…O2 | 0.889 | 2.436 | 2.807 | 105.41 |
| N3-H…O3 | 0.889 | 2.201 | 3.074 | 166.92 |
| N3-H…O4 | 0.889 | 2.367 | 2.767 | 107.44 |
| N3-H…O5 | 0.890 | 2.111 | 3.000 | 176.86 |
| N3-H…O6 | 0.890 | 2.454 | 2.830 | 105.90 |
| 296K | | | | |
| N1-H…O7 | 0.890 | 1.873 | 2.864 | 179.38 |
| N1-H…O8 | 0.890 | 2.539 | 2.943 | 108.29 |
| N1-H…O9 | 0.891 | 1.952 | 2.842 | 176.98 |
| N1-H…O10 | 0.889 | 2.537 | 2.952 | 109.24 |
| N1-H…O11 | 0.889 | 1.930 | 2.809 | 169.22 |
| N1-H…O12 | 0.890 | 2.612 | 2.993 | 106.76 |
| N3-H…O1 | 0.890 | 2.070 | 2.939 | 165.23 |
| N3-H…O2 | 0.890 | 2.497 | 2.896 | 107.75 |
| N3-H…O3 | 0.890 | 2.052 | 2.936 | 172.43 |
| N3-H…O4 | 0.890 | 2.612 | 2.967 | 104.80 |
| N3-H…O5 | 0.890 | 1.991 | 2.879 | 175.09 |
| N3-H…O6 | 0.890 | 2.485 | 2.878 | 107.27 |

Table S2. Hydrogen-bond geometry(**Å,** deg) in between phosphoric acid and phosphate radical for compound **1**.

| D-H…A | d(D-H) **Å** | d(H…A) **Å** | d(D…A) **Å** | D-H…A(°) |
| --- | --- | --- | --- | --- |
| 100K | | | | |
| O20-H…O33 | 0.821 | 1.841 | 2.569 | 147.11 |
| 018-H…O26 | 0.820 | 1.921 | 2.568 | 150.96 |
| 019-H…028 | 0.820 | 1.819 | 2.584 | 154.48 |
| O23-H…O29 | 0.822 | 2.103 | 2.484 | 108.11 |
| O30-H…O22 | 0.819 | 1.893 | 2.472 | 126.79 |
| O24-H…O25 | 0.821 | 1.793 | 2.586 | 162.19 |
| O21-H…O28 | 0.820 | 1.831 | 2.546 | 172.94 |
| O26-H…O33 | 0.820 | 1.789 | 2.492 | 142.73 |
| O32-H…O17 | 0.819 | 1.711 | 2.485 | 156.72 |
| O30-H…O22 | 0.819 | 1.893 | 2.472 | 126.79 |
| O31-H…O36 | 0.819 | 1.926 | 2.503 | 139.37 |
| O1W-H…O29 | 0.961 | 1.979 | 2.839 | 179.59 |
| O1W-H…O34 | 0.959 | 2.234 | 2.721 | 118.57 |
| O34-H…O36 | 0.849 | 1.692 | 2.436 | 172.40 |
| O35-H…O25 | 0.820 | 1.826 | 2.619 | 162.24 |
| 296K | | | | |
| O24-H…O25 | 0.821 | 1.782 | 2.672 | 160.91 |
| O21-H…O28 | 0.819 | 1.831 | 2.635 | 166.83 |
| O19-H…028 | 0.821 | 2.109 | 2.612 | 110.08 |
| O27-H…O1W | 0.810 | 2.135 | 2.673 | 123.93 |
| O26-H…O33 | 0.821 | 1.799 | 2.692 | 141.14 |
| O32-H…O17 | 0.820 | 1.744 | 2.519 | 156.89 |
| O20-H…O33 | 0.820 | 1.875 | 2.598 | 146.41 |
| 018-H…O26 | 0.820 | 1.935 | 2.651 | 145.41 |
| O31-H…O36 | 0.821 | 1.907 | 2.598 | 141.09 |
| O30-H…O22 | 0.819 | 1.826 | 2.583 | 136.28 |
| O23-H…O29 | 0.820 | 1.940 | 2.552 | 119.65 |
| O35-H…O25 | 0.819 | 1.842 | 2.609 | 155.38 |
| O34-H…O36 | 0.820 | 3.108 | 2.626 | 39.21 |
| O1W-H…O34 | 0.820 | 2.077 | 2.866 | 161.35 |
| O1W-H…O29 | 0.931 | 2.302 | 3.169 | 154.98 |

Table S3. Structure parameters for the 18-crown-6 rings in compound **1**

|  | RTP-A | RTP-B | LTP-A | LTP-B |
| --- | --- | --- | --- | --- |
| Bond distance, **Å** | | | | |
| average C-C | 1.458 | 1.474 | 1.490 | 1.498 |
| range of C-C | 1.356-1.560 | 1.449-1.511 | 1.484-1.495 | 1.482-1.511 |
| Bond angles, deg | | | | |
| average C-O-C | 114.56 | 113.05 | 112.73 | 113.26 |
| range of C-O-C | 110.88-123.37 | 109.66-115.57 | 111.51-113.82 | 112.53-114.57 |
| average C-C-O | 112.26 | 108.58 | 108.77 | 108.42 |
| range of C-C-O | 107.57-124.11 | 106.87-110.36 | 108.03-109.19 | 107.14-109.91 |
| Absolute values of torsion angles, deg | | | | |
|  | | | | |
| average O-C-C-O | 57.51 | 65.50 | 64.51 | 61.81 |
| range of O-C-C-O | 45.15-68.12 | 61.67-70.05 | 60.38-68.38 | 54.39-66.77 |

Table S4. Anisotropic thermal factors (Beq) of the atoms in the 3-nitroanilinium cation of compound **1** at 100K and 296K

|  | O13 | O14 | N2 | C25 | C26 | C27 | C28 | C29 | C30 | N1 |
| --- | --- | --- | --- | --- | --- | --- | --- | --- | --- | --- |
| 100K | 4.1238 | 4.0053 | 2.3937 | 1.4615 | 1.6195 | 1.6827 | 2.4016 | 2.7966 | 2.2594 | 1.5958 |
| 296K | 9.559 | 9.954 | 4.9691 | 3.6261 | 3.7525 | 3.9816 | 5. 056 | 6.162 | 4.8506 | 3.8947 |
|  | O16 | O15 | N4 | C31 | C32 | C33 | C34 | C35 | C36 | N3 |
| 100K | 4.2502 | 2.9151 | 2.4411 | 1.3983 | 1.5563 | 1.7143 | 2.1093 | 2.2357 | 1.7696 | 1.5247 |
| 296K | 10.033 | 9.638 | 6.0909 | 3.7999 | 3.634 | 4.0448 | 6.004 | 6.083 | 6.0198 | 4.266 |
